# Regulatory T-cells-related signature for identifying a prognostic subtype of hepatocellular carcinoma with an exhausted tumor microenvironment

**DOI:** 10.3389/fimmu.2022.975762

**Published:** 2022-09-15

**Authors:** Genhao Zhang

**Affiliations:** Department of Blood transfusion, The First Affiliated Hospital of Zhengzhou University, Zhengzhou, China

**Keywords:** HCC, prognosis, PD1/PD-L1, Tregs, TME, exhausted

## Abstract

Regulatory T-Cells (Tregs) are important in the progression of hepatocellular cancer (HCC). The goal of this work was to look into Tregs-related genes and develop a Tregs-related prognostic model. We used the weighted gene co-expression network analysis (WGCNA) to look for Tregs-related genes in the TCGA, ICGC, and GSE14520 cohorts and then used the non-negative matrix factorization (NMF) algorithm to find Tregs-related subpopulations. The LASSO-Cox regression approach was used to determine Tregs-related genes, which were then condensed into a risk score. A total of 153 overlapping genes among the three cohorts were considered Tregs-related genes. Based on these genes, two Tregs-associated clusters that varied in both prognostic and biological characteristics were identified. When compared with Cluster 1, Cluster 2 was a TME-exhausted HCC subpopulation with substantial immune cell infiltration but a poor prognosis. Five Tregs-related genes including *HMOX1, MMP9, CTSC, SDC3*, and *TNFRSF11B* were finally used to construct a prognostic model, which could accurately predict the prognosis of HCC patients in the three datasets. Patients in the high-risk scores group with bad survival outcomes were replete with immune/inflammatory responses, but exhausted T cells and elevated PD-1 and PD-L1 expression. The results of qRT-PCR and immunohistochemical staining (IHC) analysis in clinical tissue samples confirmed the above findings. Moreover, the signature also accurately predicted anti-PD-L1 antibody responses in the IMvigor210 dataset. Finally, *HMOX1*, *MMP9*, and *TNFRSF11B* were expressed differently in Hep3B and Huh7 cells after being treated with a PD1/PD-L1 inhibitor. In conclusion, our study uncovered a Tregs-related prognostic model that could identify TME- exhausted subpopulations and revealed that PD1/PD-L1 inhibitors could alter the expression levels of *HMOX1*, *MMP9*, and *TNFRSF11B* in Hep3B and Huh7 cells, which might help us better understand Tregs infiltration and develop personalized immunotherapy treatments for HCC patients.

## Introduction

Chronic liver inflammation caused by HBV or HCV infection, alcoholism, or nonalcoholic fatty liver disease (NAFLD) can result in an aberrant concentration of immune cells in tumors and adjacent tissues, including T lymphocytes, macrophages, and dendritic cells (1). These immune cells, together with other non-immune components (fibroblasts, extracellular matrix), comprise the tumor microenvironment (TME) surrounding cancer cells (2). The dynamic interaction between cancer cells and TME can disrupt tumor cells’ immune surveillance, accelerate tumor cell proliferation, clonal evolution, immune evasion, and treatment resistance, and play a key role in tumor genesis and progression (3). TME potentially causes tissue remodeling and functional impairment by generating local hypoxia in tumor tissue and ultimately promoting tumor metastasis (4). In addition, TME can impact the delivery of anticancer medications to the tumor location by interacting with mesenchymal stem cells (5). CD4^+^CD25^+^FoxP3^+^ T regulatory lymphocytes (Tregs), as an important heterogeneous T cell subset, have been identified to participate in the development of HCC by promoting immune tolerance (6). Treg cells are abundant in tumors and can make up 10 to 50 percent of the CD4^+^ T cells there (7). Notably, the proportion of Treg cells in the peripheral blood did not match the density of Treg cells in the TME, indicating that the study of the function of Treg cells in the TME is more crucial in the field of cancer immunology. Depletion of T-reg cells encourages the growth of high endothelial venules, which are crucial for lymphocyte recruitment (8). Tregs can block cytotoxic CD8^+^ T lymphocytes (CTLs) cytotoxic and proliferative capabilities, aid in the creation of an immunosuppressive TME, and are linked to the advancement of HCC (9), while CD8^+^ T cells specific for neoantigens are more resistant to Treg cell-mediated immune suppression (10). Crosstalk between Tregs and neutrophil extracellular traps promotes the transition of NAFLD to HCC (11). TGFβ-activated stromal cells reduce the recruitment of Tregs in TME, thereby regulating the balance between anti-tumor and pro-tumor immune cells (12). High Tregs infiltration is also linked to poor outcomes and recurrence in HCC patients (13, 14). In recent years, immunotherapy using immune checkpoint inhibitors (ICIs), such as anti-PD1 antibody nivolumab and anti-PD-L1 agents atezolizumab, has shown strong antitumor activity in a subset of HCC patients by blocking the interaction of PD1 with its ligands, thereby preventing exhaustion or dysfunction of effector T cells (15). Interestingly, there is a close and complex relationship between Tregs infiltration and PD1 expression. Tregs expressing PD1 in the TME can impact immunosuppressive function and are associated with progression in cancer patients (16). PD1 blockade induces enhanced PD1^+^ Tregs-mediated immunosuppression (17). In addition, Lenvatinib can improve anti-PD1 effectiveness by reducing tumor PD-L1 levels and Tregs differentiation (18). Therefore, exploring them more deeply can help us gain a deeper understanding of the complex mechanisms of the TME in HCC development, and help clinicians to formulate strategies for the use of ICIs in cancer treatment.

Given the difficulty in collecting enough tumor tissue for tumor-infiltrating lymphocyte (TIL) assay analysis by flow cytometry (FCM) and the fact that crosstalk between Tregs and cancer is a complex process involving multiple genes, we built and validated a prognostic stratification model based on Tregs-related genes in public datasets that could be used to efficiently categorize HCC patients prognostically and predict their response to anti-PD-L1 immunotherapy.

## Materials and methods

### Acquisition of public datasets and clinical sample processing

Transcriptome expression data from HCC patients were gathered from three public databases, including the TCGA-LIHC (2022.04), the ICGC (LIRI-JP, 2019.11), and the GSE14520 (2010.12) cohorts. The clinical characteristics of HCC patients in the three cohorts were displayed in **Table S1**. Patients with incomplete survival data or who lived for less than one month were eliminated from the study. Clinically verified samples for qRT-PCR research were fresh frozen tumor biopsies and their surrounding normal tissues from 20 previously collected HCC patients. The primer sequences are shown in **Table S2**. Zhengzhou University’s Ethics Committees gave its approval to this work. Written informed consent was taken.

### Estimation of immune cell infiltration and TME scores

The relative abundance of 28 immune cell subtypes in the three datasets was assessed by the ssGSEA algorithm, and the immune cell abundance identifier (ImmuCellAI, 2020.02) was further utilized to specifically assess the abundance of comprehensive T cell subsets (19). ESTIMATE algorithm was used to calculate stromal and immunological scores in tumor tissue based on gene expression patterns of HCC samples to determine the quantity of stromal and immune cells inside the tumor (20). Immunohistochemical staining (IHC) was performed to explore the infiltration of Tregs and CD8^+^ T cells in HCC tissues. Two competent pathologists performed IHC findings assessment using a single-blind and uniform standard procedure.

### Tregs-related genes identification by the weighted gene co-expression network analysis

The WGCNA was used to create a scale-free co-expression network based on transcriptome expression data from the three datasets to find the gene modules most relevant for Tregs infiltration abundance. Standard deviation (SD > 50%) was used to screen for highly variable genes. Module membership represented the link between module characteristic genes and gene expression patterns, whereas gene significance (GS) was utilized to assess the relationship between individual genes and Tregs infiltration abundance. The genes identified from the modules most linked with Tregs infiltration abundance were appraised as candidate genes using a p-value threshold of GS < 0.0001 and a significance level of univariate Cox regression of p < 0.01. The overlapping genes of the candidate genes in the three datasets were finally confirmed as Tregs-related genes and used for subsequent analysis.

### Identification of prognostic molecular subtypes by the non-negative matrix factorization algorithm

Based on the Tregs-related genes obtained above, patients were clustered using the NMF algorithm, the standard was “brunet”, and the iterations were 50 times. The number of clusters varies from 2 to 6, and the optimal number of clusters is determined based on cophenetic, dispersion, and contour. Kaplan-Meier survival analyses were further performed to assess differences in patients’ survival rates between different subtypes.

### DEG identification and functional enrichment analysis

Gene Expression Profile Interaction Analysis (GEPIA, 2017.07) (21) (http://gepia.cancer-pku.cn/) was used to investigate the expression levels of Tregs-related genes, and genes with statistically significant differences were defined as differentially expressed genes (DEGs) with a P-value < 0.05 and a |log_2_FC| cutoff criterion of ≥0.5. The Metascape database (22) (http://metascape.org/, 2019.04) was then used to investigate the functional annotation of DEGs for GO and KEGG pathway analysis. With a significant threshold of |normalized enrichment score|>1 and a nominal p-value of < 0.05, Gene set enrichment analysis (GSEA) was used to investigate changes in Hallmarks.

### Formation and validation of Tregs-related prognostic risk scoring model

The correlation between Tregs-related genes and HCC patient survival outcomes was calculated using univariate Cox regression with a P-value < 0.01. The Tregs-related genes with prognostic significance were then investigated using the LASSO-Cox regression technique and a classifier linked with prognosis was established. The multivariate Cox relapse coefficient (β) was used to create a risk score based on the concept of directly mixing the equation below with the mRNA expression level. The risk score = ∑iCoefficient (mRNAi)*Expression (mRNAi). Due to the optimal hazard score edge, we divided the HCC patients into two categories. ROC analysis, Kaplan-Meier survival analysis, and cox relative risks relapse investigation were used to assess the prognostic signature’s predictive autonomy. The ICGC and GSE14520 datasets were used as validation cohorts for validating our constructed Tregs-related signature.

### Genetic alterations and drug susceptibility analysis

The R package “maftools” was used to assess the differences in genetic variations between various subgroups using the mutation and CNA data of 342 HCC patients acquired from the TCGA dataset. The association between anticancer drug sensitivity and mRNA molecules in our risk model was directly explored in the CellMiner database (2012.07) (23) with cutoff criteria of adjusted P-values <0.001 and Pearson’s correlation coefficients >0.4.

### Immunohistochemistry staining analysis

The paraffin samples were cut into 4 μm slides and soaked for the identification of Tregs and CD8^+^ T cells. The tissue fragments were progressively hydrated in graded alcohol after being deparaffinized in xylene. By heating 0.01 mol/L citrate buffer in a steam cooker for 10 minutes, antigen retrieval was accomplished. To suppress endogenous peroxidase activity, slides were washed with PBS and then incubated for 20 minutes at 37°C with a 0.3 percent H_2_O_2_ solution. Slides were then blocked with bovine serum albumin (BSA), and continuously incubated with anti-FOXP3 and CD8 antibodies overnight at 4°C, respectively. After being washed with PBS, slides were continuously incubated with secondary antibodies coupled to horseradish peroxidase (HRP) for 30 minutes. Utilizing HRP’s routine substrate detection, immune complexes were found. Slides were then dehydrated in graded alcohols and xylene after being stained with hematoxylin.

### Cell culture and PD1/PD-L1 inhibitor treatment

Hep3B and Huh7 cells from the Cell Bank of the Shanghai Institute of Cell Research, Chinese Academy of Sciences (Shanghai, China) were cultured in the suggested DMEM medium (Sangon Biotech, China) containing 10% fetal bovine serum (FBS, Sangon Biotech, Shanghai, China) at 100% humidity, 37°C, and 5% CO2. Cells were incubated for 4 hours at room temperature in the DMEM medium containing 4 mg/mL PD1/PD-L1 inhibitor (Abcam, ab230369, UK) for PD1/PD-L1 blockade.

### Statistical analysis

Categorical data were compared using Pearson’s chi-square test or Fisher’s exact test when appropriate, and quantitative data between two groups were compared using the t-test. The examination of data from three or more groups was done using a one-way analysis of variance (ANOVA). R software (Version 4.0.3) was used to analyze the prediction performance of survival outcomes using receiver operating characteristic (ROC) curve analysis and Kaplan-Meier survival analysis. The association between a prognostic classifier and survival outcomes was investigated using a Cox proportional model. When the P-value < 0.05, the results were considered statistically significant. The flowchart of this study is shown in **Figure S1**.

## Results

### Identification genes associated with Tregs infiltration

**Figure 1:**
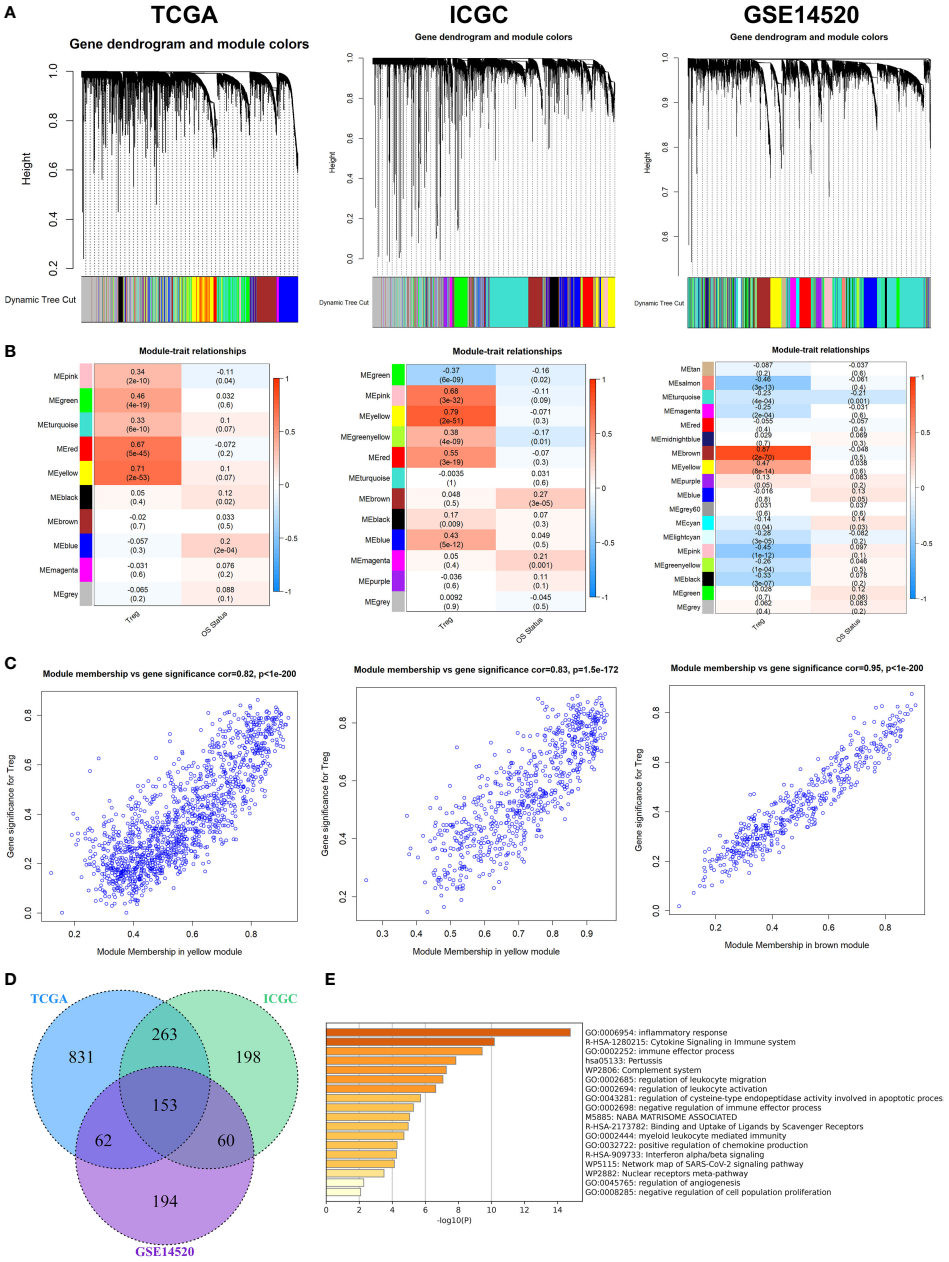
WGCNA for Tregs-related genes. **(A)** The coexpression network was established in the TCGA, the GSE14520, and the ICGC cohorts. **(B)** Heatmap demonstrating the correlation between module eigengenes and Tregs. **(C)** Determination of modules most significantly associated with Tregs infiltration. **(D)** 153 overlapping genes among the three cohorts were identified as Tregs-related genes. **(E)** The biological importance of these Tregs-related genes was mainly enriched in the immune-inflammatory response and regulation of lymphocytes.

After removing outliers (**Figure S2**), 9, 11, and 17 non-grey modules were created in the three datasets, respectively, according to the results of WGCNA (**Figure 1A**). As shown in **Figures 1B, C**, the yellow module was the most significantly related to Tregs infiltration in the TCGA cohort (R^2^ = 0.82, P = 2e−53), and the yellow module was the most significantly related to Tregs infiltration in the ICGC cohort (R^2^ = 0.83, P = 2e−51), and the brown module was the most significantly related to Tregs infiltration in the GSE14520 cohort (R^2^ = 0.95, P = 2e−70), respectively. 153 overlapping genes among the three cohorts were identified as Tregs-related genes (**Figure 1D**). The biological importance of these Tregs-related genes was mainly enriched in the immune-inflammatory response and regulation of lymphocytes (**Figure 1E**). When expression in normal tissues was considered, out of 153 genes, only 16 were identified as differentially expressed genes (DEGs) (**Figure S3A**). Then, a PPI network was performed to explore the potential interactions between these DEGs (**Figure S3B**), and the biological importance of these Tregs-related DEGs was mainly enriched in Cytokine signaling and regulation of leukocytes (**Figure S3C**).

### Identification of prognostic molecular subtypes

**Figure 2:**
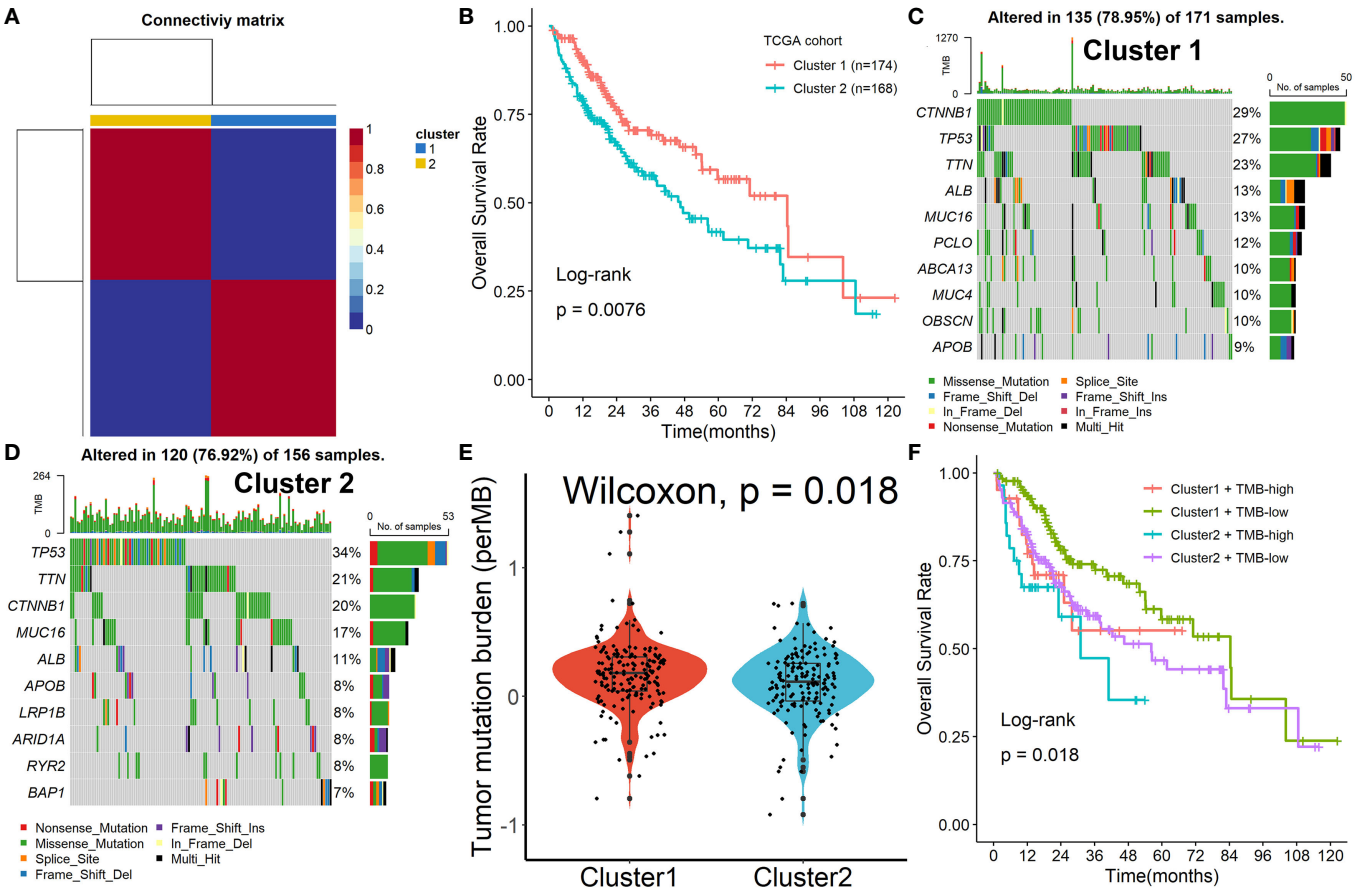
Identification of prognostic molecular subtypes by the NMF algorithm. **(A)** Number two was identified as the optimal number of clusters. **(B)** Patients in Cluster 2 had better survival outcomes when compared with patients in Cluster 1. **(C, D)** The mutation rates of mutated genes in the two subgroups. **(E)** Patients in Cluster 1 had a higher TMB value, compared with patients in Cluster 2. **(F)** Patients in Cluster 2 with high TMB values had the worst survival outcomes when compared with others.

To further explore the mechanism of these 153 Tregs-related genes in HCC, the NMF algorithm was performed. Due to the cophenetic, dispersion, and profile (**Figure S4**), number two was identified as the optimal number of clusters (**Figure 2A**). Patients in Cluster 2 had better survival outcomes when compared with patients in Cluster 1 (**Figure 2B**). Subsequently, we found that the mutation rates of mutated genes in the two subgroups were also significantly different. The most commonly mutated gene in the Cluster 1 was CTNNB1 (29%, **Figure 2C**), while it was TP53 (34%, **Figure 2D**) in Cluster 2. After the tumor mutation burden (TMB) was estimated, patients in Cluster 1 had a higher TMB value, compared with patients in Cluster 2 (**Figure 2E**). Finally, we found that patients in Cluster 2 with high TMB values had the worst survival outcomes when compared with others (**Figure 2F**).

### Patients in Cluster 2 had an exhausted immune microenvironment

**Figure 3:**
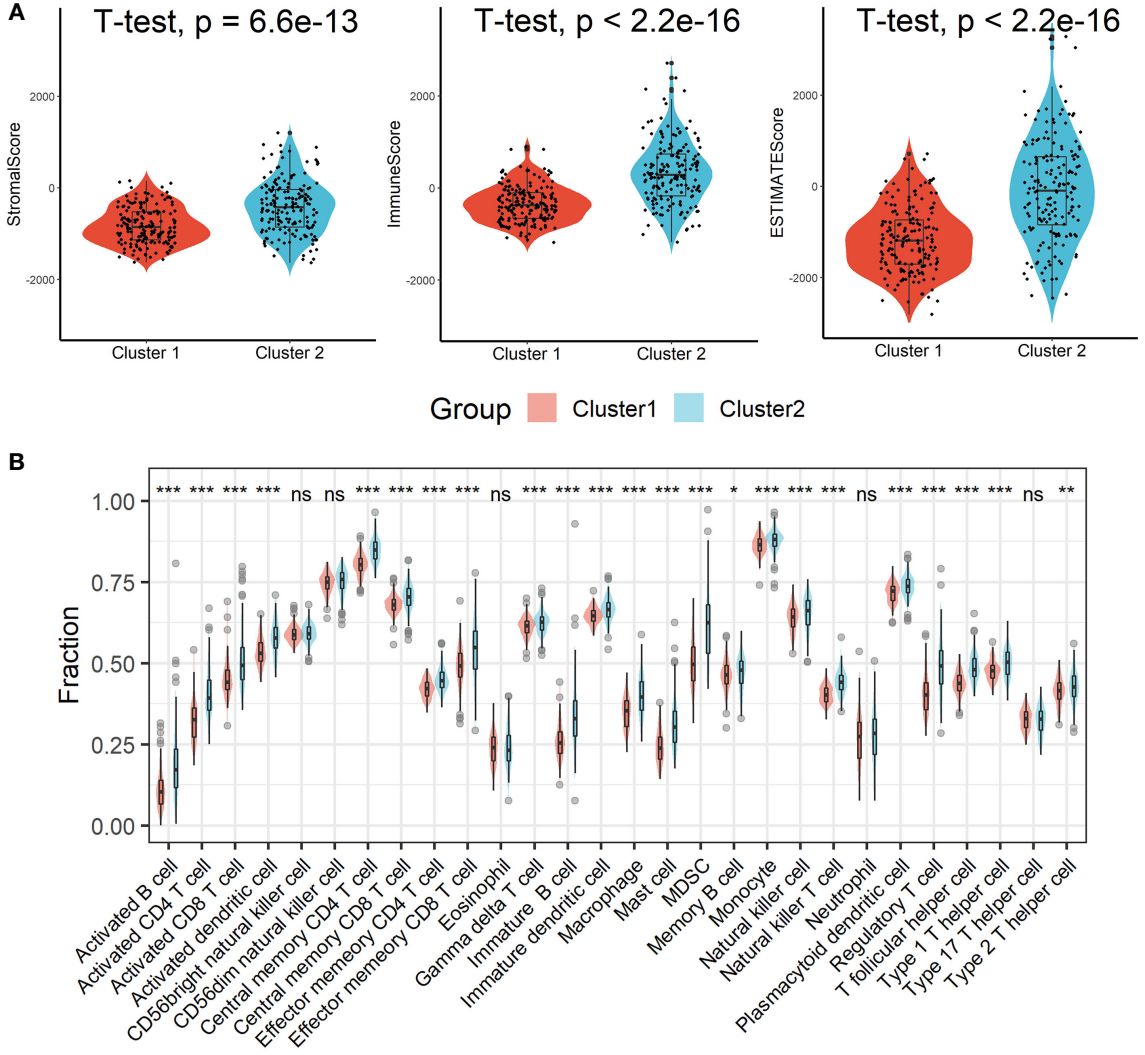
Estimation of immune cell infiltration in different clusters. **(A)** Patients in Cluster 2 had higher immune, stromal, and ESTIMATE scores compared with patients in Cluster 1. **(B)** Almost all types of immune cells were higher in Cluster 2 than those in Cluster 1 except for CD56bright natural killer cell, CD56dim natural killer cell, eosinophil, neutrophil, and Type 17 T helper cell. ns, not significant; *p < 0.05; **p <0.01; ***p < 0.001.

**Figure 4:**
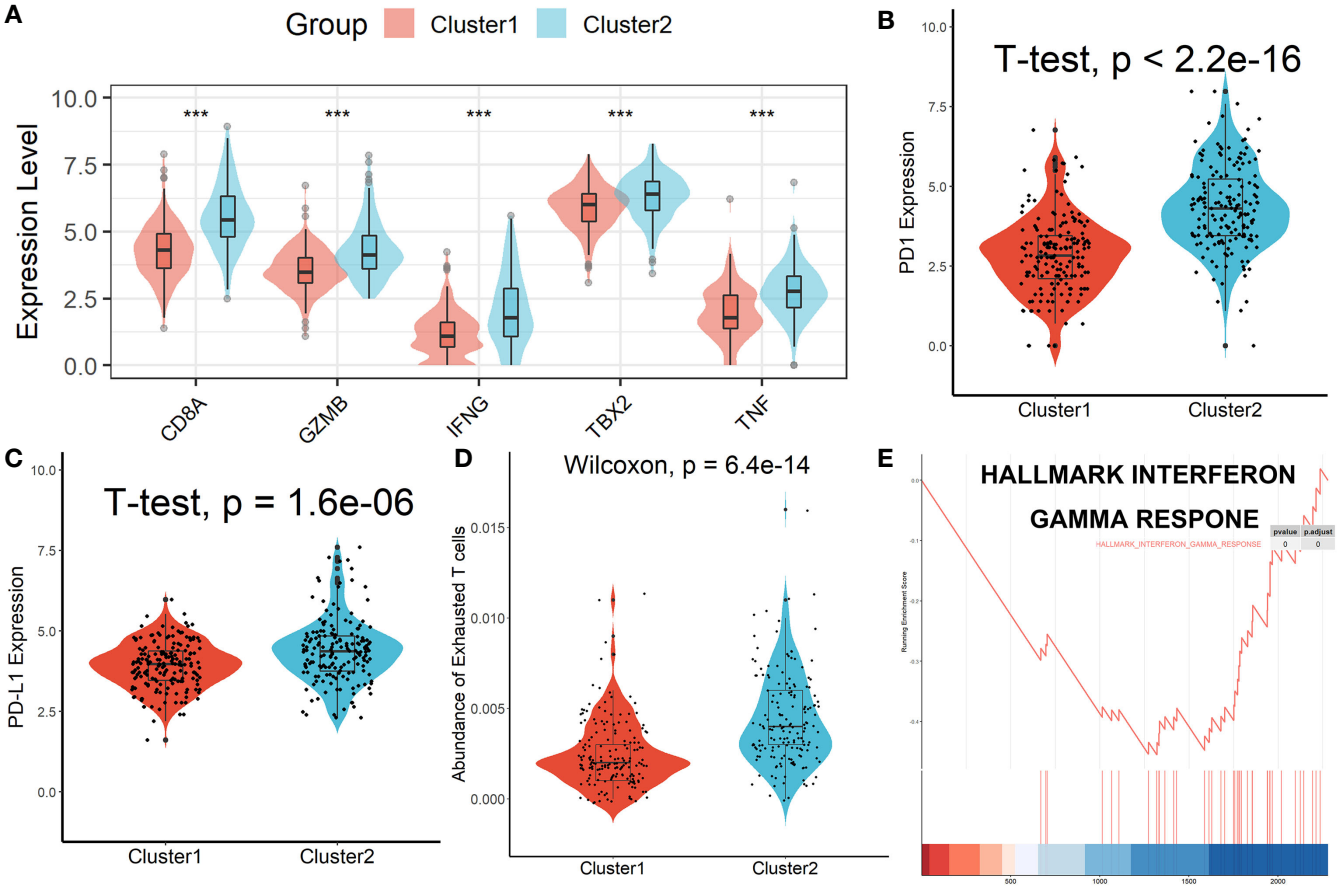
Patients in Cluster 2 had an Exhausted Immune Microenvironment. **(A)** Differential analysis of inflammation/immune response-related genes. **(B, C)** Differential analysis of PD1 and PD-L1 expression. **(D)** Exhausted T cells were significantly enriched in Cluster 2. **(E)** Cluster 1 displayed an attenuated IFN-γ response. ***p < 0.001.

Patients in Cluster 2 had higher immune, stromal, and ESTIMATE scores compared with patients in Cluster 1, as shown in **Figure 3A**. According to the ssGSEA algorithm, almost all types of immune cells were higher in Cluster 2 than those in Cluster 1 except for CD56bright natural killer cell, CD56dim natural killer cell, eosinophil, neutrophil, and Type 17 T helper cell (**Figure 3B**). Interestingly, we found that patients in Cluster 2 had poor survival outcomes but a higher abundance of CD8^+^ T cells infiltration. Considering that decreased infiltration levels of CD8^+^ T cells were often associated with poor survival rates, therefore, we assumed that these CD8^+^ T cells in Cluster 2 were exhausted T cells. To test this conjecture, we then analyzed genes involved in immune/inflammatory responses, including *CD8A*, *GZMB*, *IFNG*, *TBX2*, and *TNF*, and found that these genes were significantly up-regulated in Cluster 2 (**Figure 4A**). We also found that the expression of PD1, a marker of exhausted T cells, was also significantly up-regulated in Cluster 2 (**Figure 4B**), as was the expression level of PD-L1 (**Figure 4C**). In addition, exhausted T cells were significantly enriched in Cluster 2, according to the results of ImmuCellAI analysis (**Figure 4D**). Finally, the GSEA results indicated that Cluster 1 displayed an attenuated IFN-γ response (**Figure 4E**), which can directly increase PD-L1 expression and activate the PD-1/PD-L1 signaling axis. Together, the aforementioned findings showed that Cluster 2 had a robust immunological and inflammatory response, but the elevated PD1 and PD-L1 in this group might result in an exhausted TME and eventually have a negative impact on the survival of HCC patients.

### Formation of Tregs-related prognostic signature in HCC

**Figure 5:**
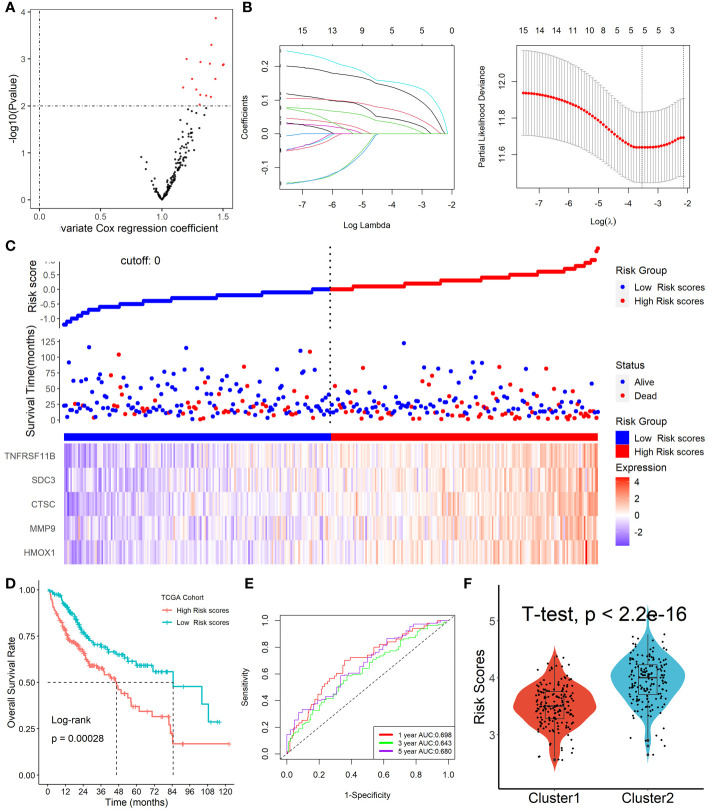
Formation of Tregs-related prognostic signature in HCC. **(A)** 15 prognosis-associated genes were identified by univariate Cox regression with a p-value less than 0.01. **(B)** Tregs-related genes were screened by the LASSO-Cox regression model. **(C)** Patients were divided into high- or low-risk scores subgroups with an optimal threshold after patients’ risk scores were calculated with the above formula. **(D)** Patients with lower risk scores were remarkably relevant to better survival outcomes. **(E)** This Tregs-related signature had a good prognostic performance. **(F)** Patients in Cluster 2 had higher risk scores compared with patients in Cluster 1.

Among the 153 overlapping genes in the three cohorts obtained by WGCNA, 15 prognosis-associated genes were identified by univariate Cox regression with a p-value less than 0.01 (**Figure 5A**). These genes were then selected by using the LASSO-Cox regression model based on the minimum value of λ (**Figure 5B**). Five genes including HMOX1, MMP9, CTSC, SDC3, and TNFRSF11B were screened out and were then put into a multivariate Cox proportional model, andfinally, a prognostic Tregs-related signature was formatted. Risk score = (0.16468758×*HMOX1*) + (0.04918601×*MMP9*) + (0.16592365×*CTSC*) +(0.06017538×*SDC3*) + (0.09164677×*TNFRSF11B*). Patients were divided into high- or low-risk scores subgroups with the median of scores after patients’ risk scores were calculated with the above formula (**Figure 5C**). We found that patients with lower risk scores were remarkably relevant to better survival outcomes (**Figure 5D**) and this Tregs-related signature had a good prognostic performance with AUCs at 1-, 3-, 5-year of 0.698, 0.643, 0.680 (**Figure 5E**). In addition, patients in Cluster 2 had higher risk scores compared with patients in Cluster 1, as shown in **Figure 5F**. Finally, after controlling for other clinical parameters, this Tregs-related signature might be used as an independent predictive factor for HCC patients (HR=2.566, 95 percent CI 1.4401 -4.5742, P = 0.0013). Only MMP9 and CSTC were significantly correlated with overall survival rates, despite the five Tregs-associated gene expression levels in the GEPIA database varied between normal and malignant tissues (**Figure S5**). Additionally, the five Tregs-related genes’ protein expression in both normal and HCC was examined in the Human Protein Atlas database (24) (HPA, www.proteinatlas.org), as shown in **Figure S6**.

### Functional enrichment and genetic alterations analysis

GSEA analysis revealed that the immunological response, controlling lymphocyte activity, and production and metabolism of cytokines were the three primary areas of changed GO and KEGG items between high- and low-risk score groups (**Figure S7**). We next discovered that the two subgroups had dramatically differing mutation rates for the affected genes. TP53 (40%) and CTNNB1 (30%) were the most frequently altered genes in the groups with high and low risk scores, respectively (**Figure S8A**). Finally, we discovered that patients with high TMB levels who had high risk scores had the lowest survival rates (**Figure S8B**).

### Verification of the Tregs-related signature in external cohorts

**Figure 6:**
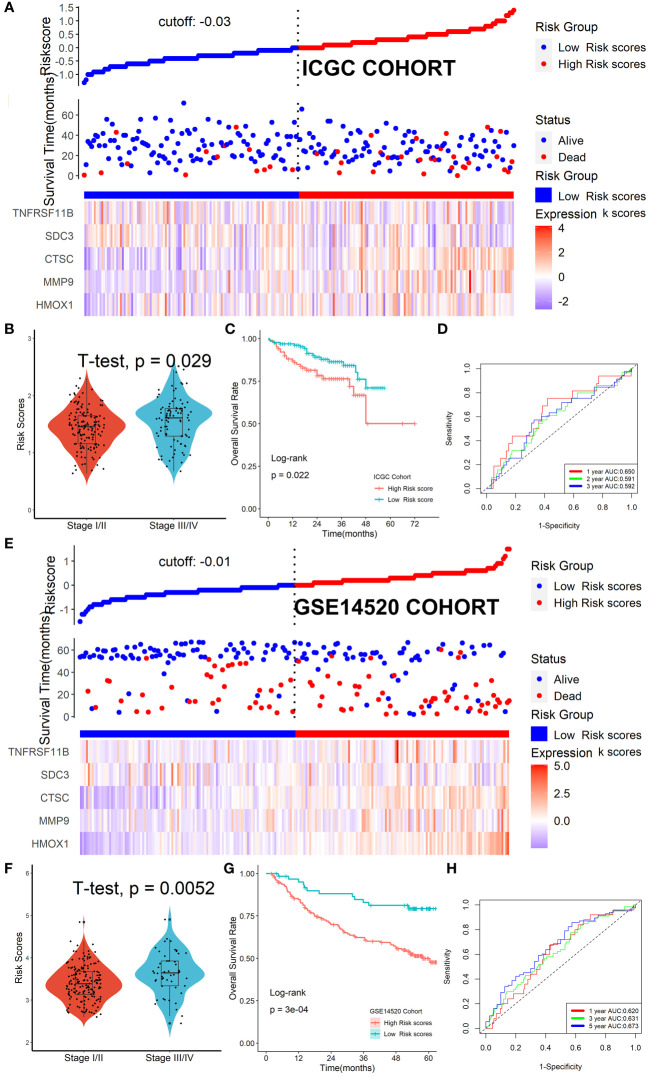
Verification of the Tregs-related signature in external cohorts. Patients were split into high- or low-risk categories in ICGC **(A)** and GSE14520 **(E)** cohorts. Patients in the later TNM stage had greater risk ratings than patients in the early stage, no matter whether we looked at the ICGC cohort **(B)** or the GSE14520 cohort **(F)**. Patients with lower risk scores were strongly associated with higher OS rates both in the ICGC cohort **(C)** and the GSE14520 cohort **(G)**. This Tregs-related signature had a good prognostic performance no matter in the ICGC cohort **(D)** or the GSE14520 cohort **(H)**.

The ICGC and GSE14520 datasets were used as validation cohorts to verify this Tregs-related signature. In the ICGC cohort (**Figure 6A**) and GSE14520 cohort (**Figure 6E**), patients’ risk scores were computed using the same formula, and patients were then split into high- or low-risk categories. No matter whether we looked at the ICGC cohort (**Figure 6B**) or the GSE14520 cohort (**Figure 6F**), we discovered that patients in the later TNM stage had greater risk ratings than patients in the early stage. Additionally, both in the ICGC cohort (**Figure 6C**) and the GSE14520 cohort (**Figure 6G**), patients with lower risk scores were strongly associated with higher OS rates. ROC analysis showed that this Tregs-related signature had a good prognostic performance with AUCs at 1-, 2-, 3-year of 0.650, 0.591, 0.629 in the ICGC cohort (**Figure 6D**) and at 1-, 3-, 5-year of 0.620, 0.631, 0.673 in the GSE14520 cohort (**Figure 6H**), respectively. Finally, after controlling for other clinical parameters, this Tregs-related signature might be used as an independent predictive factor for HCC patients in the GSE14520 cohort (HR=1.608, 95%CI 1.006-2.569, *P* = 0.046) but not in the ICGC cohort, which may be related to tumor heterogeneity.

### Patients in the high-risk score group had an exhausted immune microenvironment

**Figure 7:**
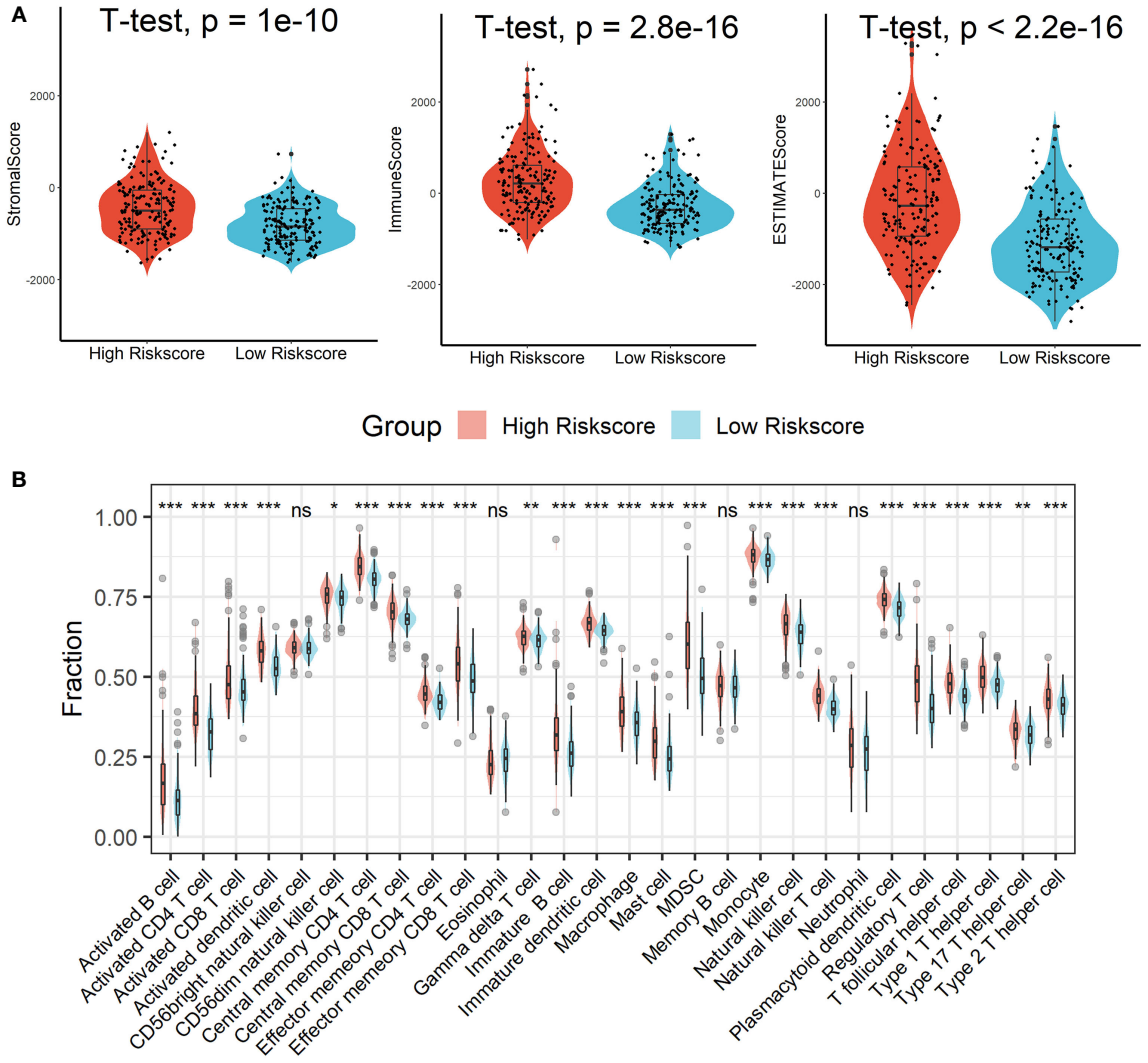
Estimation of immune cell infiltration in different risk scores groups. **(A)** Patients in the high-risk scores group had higher immune, stromal, and ESTIMATE scores compared with patients in the low-risk scores group. **(B)** Almost all types of immune cells were higher in the high-risk scores group than those in the low-risk scores group except for CD56bright natural killer cell, memory B cell, neutrophil, and eosinophil. ns, not significant; *p < 0.05; **p <0.01; ***p < 0.001.

**Figure 8:**
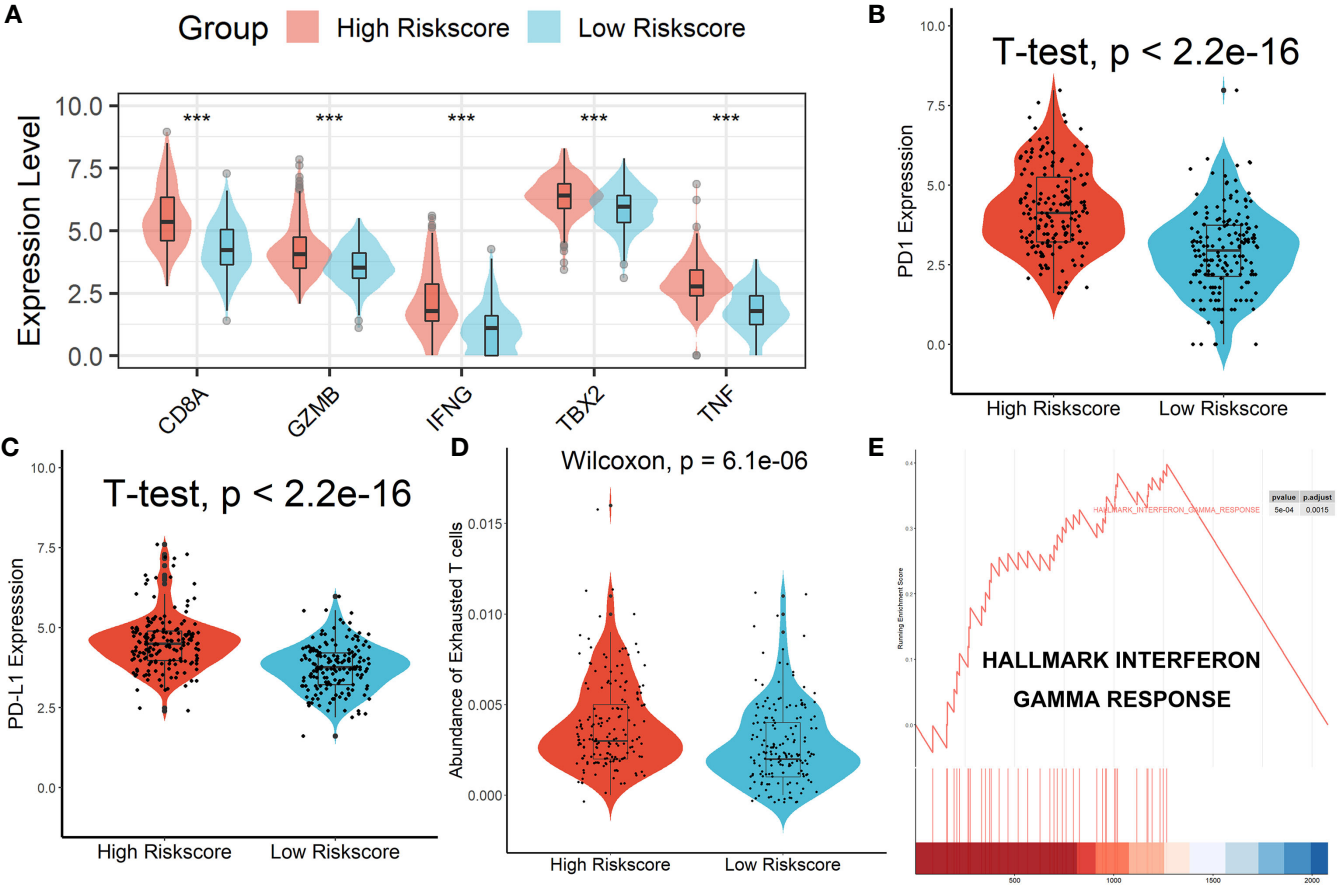
Patients in the high-risk score group had an Exhausted Immune Microenvironment. **(A)** Differential analysis of inflammation/immune response-related genes. **(B, C)** Differential analysis of PD1 and PD-L1 expression. **(D)** Exhausted T cells were significantly enriched in the high-risk scores group. **(E)** The low-risk scores group displayed an attenuated IFN-γ response. ***p < 0.001.

Patients in the high-risk scores group had higher immune, stromal, and ESTIMATE scores compared with patients in the low-risk scores group, as seen in **Figure 7A**. According to the ssGSEA algorithm, almost all types of immune cells were higher in the high-risk scores group than those in the low-risk scores group except for CD56bright natural killer cell, memory B cell, neutrophil, and eosinophil (**Figure 7B**). In addition, we also assessed the abundance of immune cell infiltration in HCC patients using various methods including TIMER (25), CIBERSORT (26), and MCP-counter (27) algorithms, and the results were consistent with the analysis results of the ssGSEA algorithm, as shown in **Figure S9**. Interestingly, we found that patients in the high-risk scores group had poor survival outcomes but a higher abundance of CD8^+^ T cells infiltration. Considering that decreased infiltration levels of CD8^+^ T cells were often associated with poor survival rates, therefore, we assumed that these CD8^+^ T cells in the high-risk scores group were exhausted T cells. To test this conjecture, we then analyzed genes involved in immune/inflammatory responses, including CD8A, GZMB, IFNG, TBX2, and TNF, and found that these genes were significantly up-regulated in the high-risk scores group (**Figure 8A**). We also found that the expression of PD1 was significantly up-regulated in the high-risk scores group (**Figure 8B**), as was the expression level of PD-L1 (**Figure 8C**). In addition, exhausted T cells were significantly enriched in the high-risk scores group, according to the results of the ImmuCellAI analysis (**Figure 8D**). Finally, the GSEA results indicated that the low-risk scores group displayed an attenuated IFN-γ response (**Figure 8E**). Together, the aforementioned findings showed that the high-risk scores group had a robust immunological and inflammatory response, but the elevated PD1 and PD-L1 in this group might result in an exhausted TME and eventually have a negative impact on the survival of HCC patients.

### Formation of a nomogram model and drug susceptibility analysis

A nomogram model was built in the TCGA dataset to investigate the coefficient prediction efficiency of this Tregs-related signature, and the results revealed that the nomogram with a C-index of 0.758 could help us provide a quantitative method for accurately predicting the 1-, 3-, and 5-year survival rate (**Figure S10A**). The calibration curves showed good agreement between the anticipated and actual probability of 1-, 3-, and 5-year survival rates (**Figure S10B**). We also uncovered 54 tumor-sensitive medications that target the five Tregs-related genes (**Table S3**), with the top 16 most important tumor-sensitive compounds indicated in **Figure S11**.

### Forecasting response to anti-PD-L1 therapy using the Tregs-related signature

**Figure 9:**
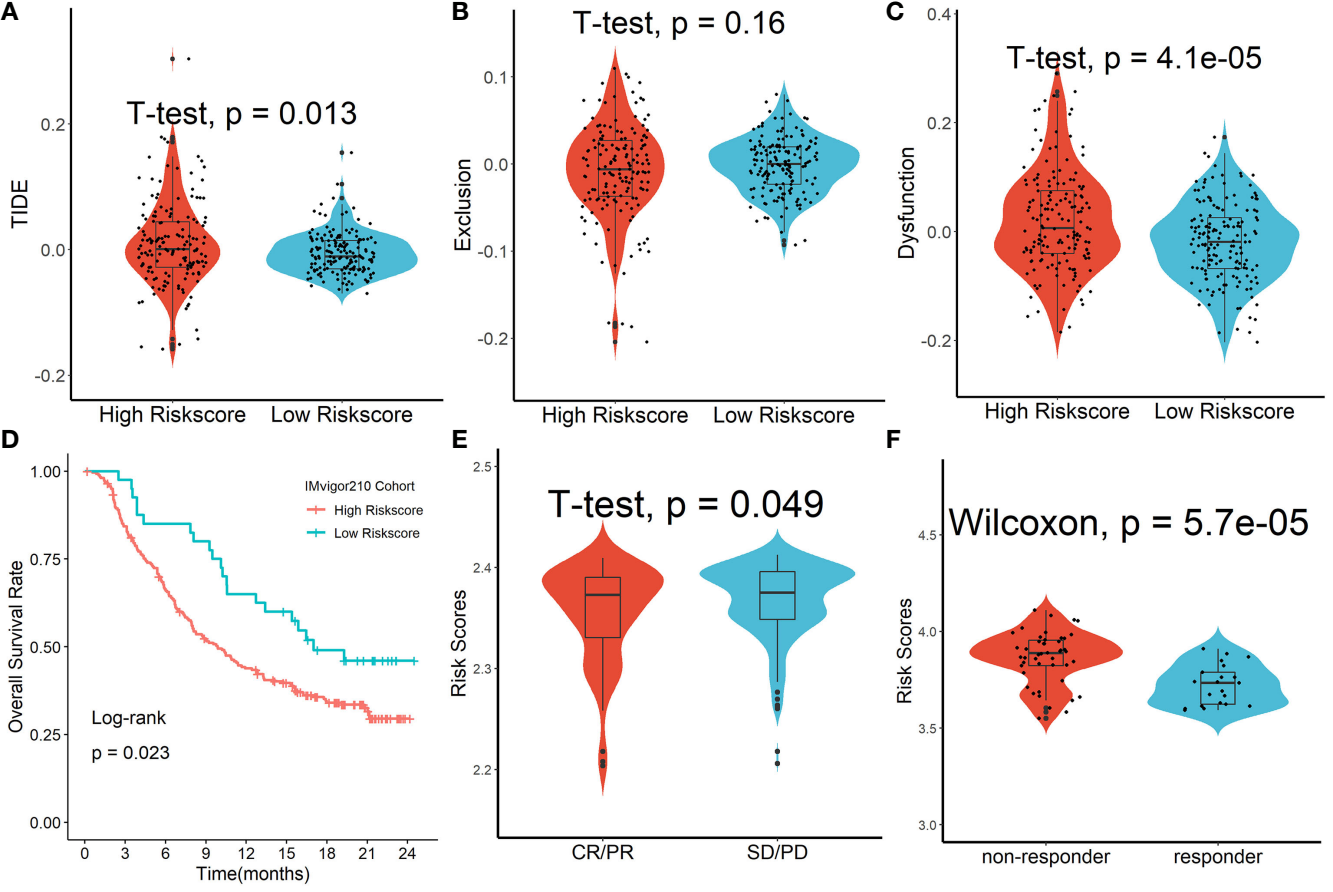
Forecasting response to anti-PD-L1 therapy using the Tregs-related signature. **(A)** TIDE scores in the high-risk scores group were much greater than that in the low-risk scores group. **(B)** T-cell exclusion scores did not differ significantly between the two groups. **(C)** T-cell dysfunction scores were greater in the high-risk scores group than in the low-risk scores group. **(D)** Patients with low risk ratings had a significant survival advantage over those with high risk scores. **(E)** Patients who had a complete response/partial response (CR/PR) had lower risk scores than patients who had stable disease/progressive disease (SD/PD). **(F)** Patients in the high-risk scores group had worse Sorafenib treatment efficacy.

We discovered that the tumor immune dysfunction and exclusion (TIDE) scores in the high-risk scores group were much greater than that in the low-risk scores group using the TIDE algorithm (**Figure 9A**). T-cell exclusion scores did not differ significantly between the two groups (**Figure 9B**), but T-cell dysfunction scores were greater in the high-risk scores group than in the low-risk scores group (**Figure 9C**). In addition, due to a shortage of data on HCC patients undergoing anti-PD-L1 medication, the IMvigor210 database was utilized as an external anti-PD-L1 cohort to investigate the possible predictive usefulness of the Tregs-related signature. This research comprised 298 individuals who exhibited an objective response. We discovered that patients with low risk ratings had a significant survival advantage over those with high risk scores (**Figure 9D**). As demonstrated in **Figure 9E**, patients who had a complete response/partial response (CR/PR) had lower risk scores than patients who had stable disease/progressive disease (SD/PD). Finally, in the GSE109211 cohort, we looked at the link between Sorafenib treatment efficacy and risk scores and discovered that patients in the high-risk scores group had worse treatment results (**Figure 9F**).

### Verification of the Tregs-related signature in clinical samples

**Figure 10:**
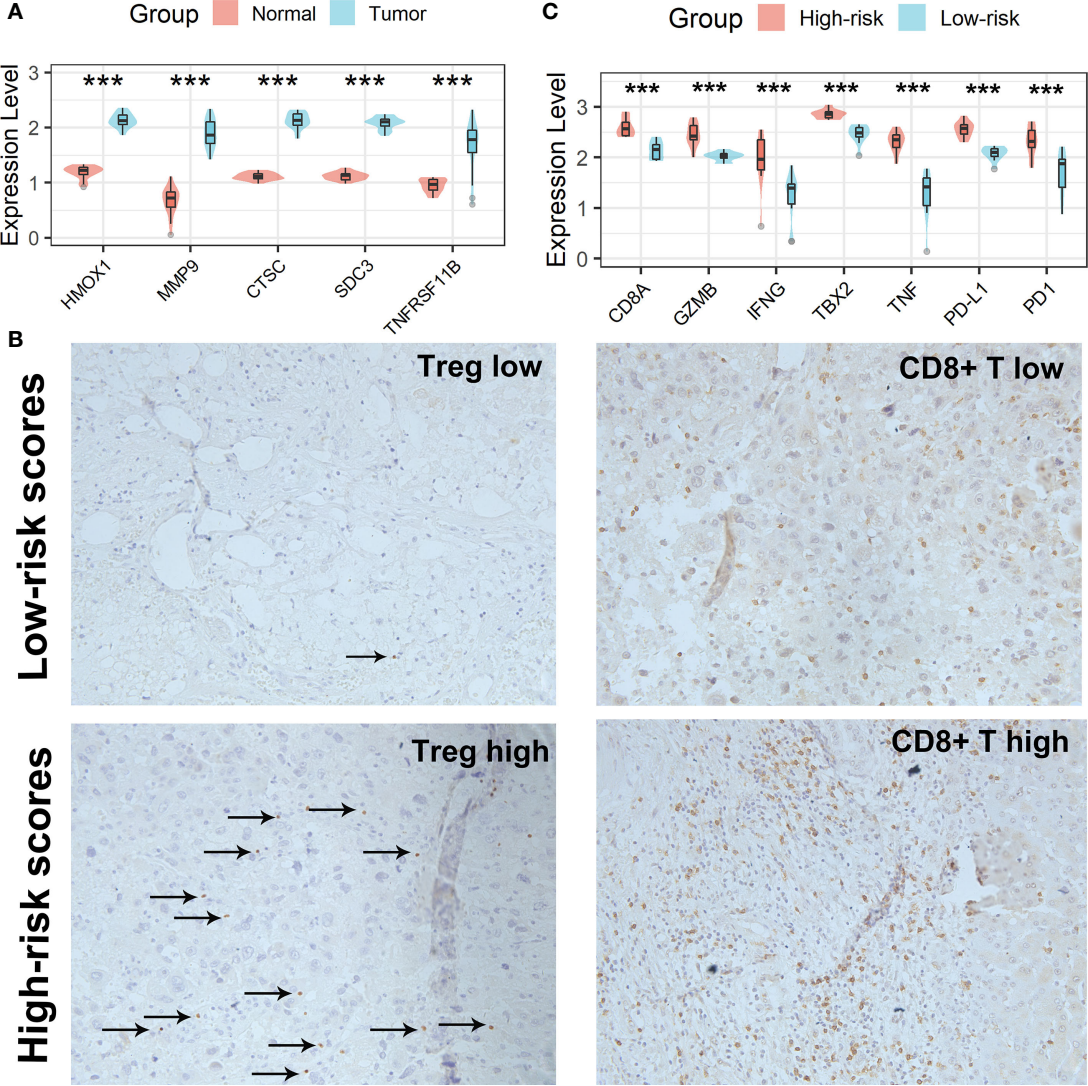
Verification of the Tregs-related signature in clinical samples. **(A)** All five Tregs-related genes revealed differential expression between normal and tumor tissues. **(B)** Patients in the high-risk scores group had a higher abundance of Tregs and CD8^+^ T cells infiltration. **(C)** Differential analysis of inflammation/immune response-related genes. ***p < 0.001.

All five Tregs-related genes revealed differential expression between normal and tumor tissues, according to the results of the qRT-PCR investigation (**Figure 10A**). After risk scores were determined using the same formula, patients were split into high-risk and low-risk groups according to the mean of the risk score. IHC was then used to analyze the infiltration of Tregs and CD8^+^ T cells in the tissues of patients in high- and low-risk scores groups. Patients in the high-risk scores group had a higher abundance of Tregs and CD8^+^ T cells infiltration (**Figure 10B**). Finally, expression levels of *CD8A*, *GZMB*, *IFNG*, *TBX2*, *TNF*, *PD1*, and *PD-L1* genes were analyzed and all of these genes were significantly up-regulated in the high-risk scores group (**Figure 10C**). Taken together, the aforementioned findings showed that the high-risk scores group had a robust immunological and inflammatory response, but the elevated PD1 and PD-L1 in this group might result in an exhausted TME and eventually have a negative impact on the survival of HCC patients.

### Verification of the Tregs-related signature after PD1/PD-L1 blockade

**Figure 11:**
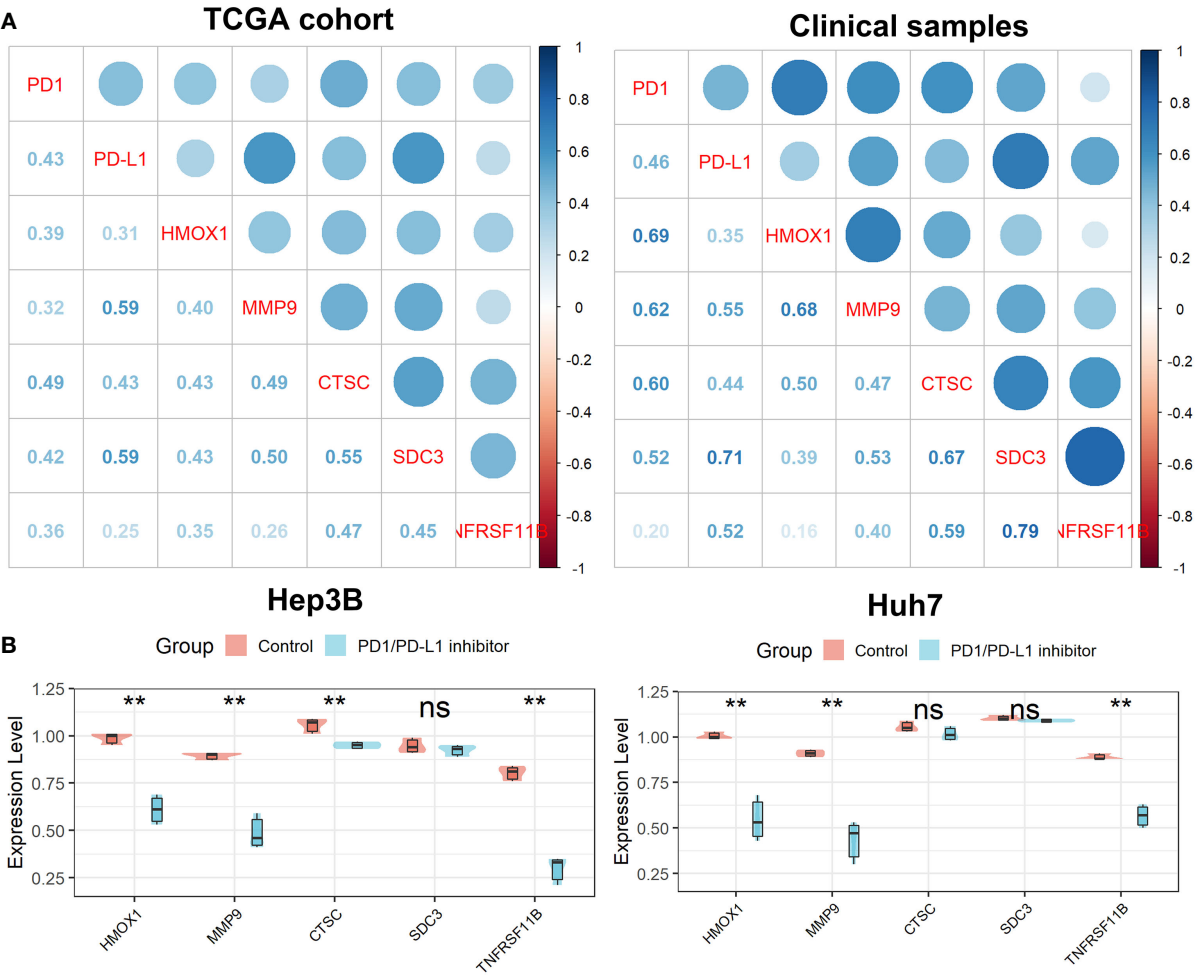
Verification of the Tregs-related signature after PD-L1 blockade. **(A)** All five Tregs-related genes were related to PD1 and PD-L1 expression. **(B)** Differential expression of the five Tregs-related genes after PD-L1 blockade. ns, not significant; **p <0.01.

As shown in **Figure 11A**, we found that all five genes in the Tregs-associated signature were significantly associated with PD1 and PD-L1 expression not only in the TCGA cohort but also in 20 clinical HCC samples, suggesting that these genes may be targets for anti-PD1/PD-L1 immunotherapy. To further explore their relationship, we treated cells with a small molecule PD1/PD-L1 inhibitor and found that the expression levels of *HMOX1*, *MMP9*, *CTSC*, and *TNFRSF11B* were significantly reduced in Hep3B cells, while only *HMOX1*, *MMP9*, and *TNFRSF11B* were expressed differently in Huh7 cells (**Figure 11B**). These confirmed our hypothesis that *HMOX1*, *MMP9*, and *TNFRSF11B* could be targeted for anti-PD1/PD-L1 immunotherapy.

## Discussion

The interaction between tumors and TME has been a hot topic in recent years (3, 28–30). On the one hand, TME plays a role in immune surveillance and immune defense of tumor cells. On the other hand, tumor-related inflammation can cause abnormal infiltration of immune cells in tumor tissue and surrounding areas, resulting in an imbalance in the production of chemokines and cytokines, helping tumor cells to adapt to immune evasion, and ultimately promote tumor development. Increasing evidence suggests that TME is involved in the occurrence and progression of HCC, the development of drug resistance, and the efficacy of immunotherapy (31, 32). Therefore, a deeper understanding of the specific mechanism of TME in HCC progression is extremely important for planning and formulating targeted therapy for HCC. According to the proportion of immune cells in the TME, HCC patients can be divided into four different subgroups: immune desert type, immunogenic type, innate immune type, and mesenchymal type (33). Among them, the immune desert type has the best prognosis due to the lack of immune cell infiltration, while the innate immune type has the worst prognosis due to the presence of a large number of immune cell infiltration and activated immune suppression. Interestingly, although cytotoxic CD8^+^ T lymphocytes (CTLs) have antitumor properties, which can induce apoptosis of tumor cells by recognizing tumor-specific antigens on target cells and releasing cytotoxic enzymes and cytokines, HCC patients with highly CTLs infiltration sometimes have poorer survival outcomes because these CTLs are exhausted (9). Various cancer cell-secreted metabolites, such as Kynurenine, S-adenosyl-L-methionine (SAM), and methylthioadenosine (MTA), have been reported to lead to T cell exhaustion (34–36). Exhaustion CD8^+^ T cells may serve as a novel biomarker for efficacy monitoring during immunotherapy in HCC patients (37). Therefore, reducing the proportion of exhausted T cells in the TME or relieving the exhausted state of T cells may become the next frontier of HCC immunotherapy.

In this research, after the relative abundance of 28 immune cell subtypes was assessed by the ssGSEA algorithm in the TCGA, ICGC, and GSE14520 cohorts, we used WGCNA to create a scale-free co-expression network to find the gene modules most relevant for Tregs infiltration abundance. The overlapping genes of the candidate genes in the three datasets were finally confirmed as Tregs-related genes and mainly enriched in the immune-inflammatory response and regulation of lymphocytes. Based on these Tregs-related genes, we divided patients into two clusters with differences in survival rates and mutation rates of mutated genes using the NMF algorithm. Compared with patients in Cluster 1, patients in Cluster 2 not only had higher immune, stroma, and estimated scores but also had higher proportions of almost all types of immune cells. Interestingly, we found that CD8+ T cells in Cluster 2 were exhausted T cells and subsequently confirmed this finding by analyzing the expression levels of PD1, PD-L1, and genes involved in immune/inflammatory responses and performing GSEA enrichment analysis. To further explore the specific mechanisms of these Tregs-related genes in HCC, we constructed a Tregs-related prognostic score model using LASSO-Cox regression. The model can not only stratify the prognosis of HCC patients well, but also effectively predict the 1-, 3-, and 5-year survival rates of patients. This Tregs-related signature was also verified in external ICGC and GSE14520 datasets. In addition, we found that CD8+ T cells in the high-risk scores group were exhausted T cells and subsequently confirmed this finding by analyzing the expression levels of PD1, PD-L1, and genes involved in immune/inflammatory responses and performing GSEA enrichment analysis. Finally, the Tregs-related prognostic score model was verified in collected fresh frozen tumor biopsies and their surrounding normal tissues by qRT-PCR and IHC analysis. Excitingly, we also observed an exhausted state of T cells in the tissue of HCC patients with high risk scores.

As a target gene of miRNA-15a-3p, heme oxygenase 1 (HMOX1) may play a role in the development and progression of HCC and is strongly correlated with the poor prognosis of HCC patients (38). HMOX1 worked in conjunction with genes involved in iron metabolism and the hypoxia phenotype to forecast patient outcomes and the effectiveness of immunotherapy (39, 40). In addition, HMOX1 has been linked to the recurrence of cancer in rats following ischemic liver transplantation (41) and can inhibit the immunomodulatory effect of Treg cells through carbon monoxide produced during metabolism (42). HMOX1 inhibitors enhance the anti-tumor effects of anti-PD-L1 antibodies in mouse melanoma and also reduce tumor size by abolishing resistance to anti-PD1 immunotherapy in female mice bearing E0771 mammary tumors (43). HMOX1 has also been implicated in PD1-involved exhausted T-cell metabolic regulation in melanoma (44). The overexpressed receptor tyrosine kinase c-Mett in renal cancer cells can inhibit cancer cell apoptosis by regulating the synergistic effect between HMOX1 and PD-L1 (45). The poor prognosis of NAFLD patients as well as HCC patients is impacted by MMP9 overexpression (46–48). Additionally, MMP9 can work with several signaling pathways to encourage the development and spread of HCC (49–52). As a crucial cytokine, MMP9 can play a role in the control of the Th17/Treg immunological imbalance (53). MMP9 was significantly positively correlated with PD-L1 and promoted poor prognosis in patients with tongue squamous cell carcinoma and colorectal cancer (54, 55). MMP9 can significantly increase PD-L1 expression by activating TGF-β-induced epithelial-to-mesenchymal (EMT) (56, 57). By raising CD8+ T cell cytotoxicity, MMP9 inhibitors can boost the therapeutic efficacy of PD-1 inhibition (58). Ayse identified significant changes in intratumoral MMP9 expression during anti-PD1 therapy in breast cancer patients using single-cell sequencing technology (59). Cathepsin C (CTSC), a lysosomal cysteine protease that is highly expressed in several tissues and a member of the papain superfamily, is essential for many biological activities. According to reports, CTSC speeds up the growth of some tumor types (60). Through the TNF-/MAPK (p38) pathway, up-regulated CTSCs in HCC have been demonstrated to promote HCC proliferation and metastasis (61). By controlling neutrophil infiltration and the development of neutrophil extracellular traps, CTSC facilitates breast cancer lung metastases (62). Gastric and colon cancer growth can be slowed down by CTSC silencing by promoting apoptosis (63, 64). A vital member of the SDC family, syndecan 3 (SDC3) is essential for cell adhesion, migration, and development. SDC3 expression is boosted by hypoxia in the tumor microenvironment, which influences pro-inflammatory reactions and the overall survival of melanoma patients (65). Additionally, SDC3 was linked to more dangerous tumors and a worse prognosis in prostate cancer (66). As a gene associated with dendritic cells, SDC3 is also important in developing a risk model for predicting the prognosis of HCC (67). The anti-apoptotic activity of TNF receptor superfamily member 11B (TNFRSF11B) can bind to and suppress TRAIL (TNF-related apoptosis-inducing ligand), which inhibits the spread of HCC and improves patient prognosis (68, 69). TNFRSF11B is significantly upregulated in peripheral blood mononuclear cells of chronic hepatitis C virus-infected patients and has been implicated in PD1-mediated T cell exhaustion and biological processes related to apoptotic signaling (70).

In this study, we found that all five genes can be acted as Treg cell-related genes to predict the prognosis and immunotherapy effect of HCC patients, and the expressions of HMOX1, MMP9, and TNFRSF11B were significantly reduced in both Hep3B and Huh7 cells after PD1/PD-L1 inhibitor treatment, suggesting that there is a certain synergy between these genes and anti- PD1/PD-L1 antibodies effect. Of course, more *in vitro* and *in vivo* studies are needed to verify the relationship and mechanism between HMOX1, MMP9, and TNFRSF11B and anti-PD1/PD-L1 therapy in HCC. In future work, we will construct a subcutaneous tumor model in C57 mice. In a nutshell, mice received subcutaneous injections of 1 x 10^5^ Hep3B and Huh7 tumor cells. After that, mice with tumors measuring 100 mm^3^ or larger were divided into four groups and given various treatments: control treatment with the PD1/PD-L1 inhibitor, treatment with HMOX1/MMP9/TNFRSF11B antibody, and the combination treatment of PD1/PD-L1 inhibitor and HMOX1/MMP9/TNFRSF11B antibody. Tumor development was monitored every three days while anti- PD1/PD-L1 therapies were given every three days and HMOX1/MMP9/TNFRSF11B antibody treatments were given every day until tumor capture on the ninth day. Finally, we observe the changes in tumor volume, the expression of inflammation-related genes, and tumor immune cell infiltration in the tumor to explore the synergistic mechanism of HMOX1/MMP9/TNFRSF11B and PD1/PD-L1 in HCC. Therefore, a deeper understanding of their mechanisms can help us dissect the complex relationship between the tumor microenvironment, the efficacy of anti-PD1/PD-L1antibody immunotherapy, and HCC.

Immune checkpoint blockade response in HCC patients can be predicted using the TIDE score. Patients in the low-risk score group had lower TIDE scores, which suggests that they may respond to ICIs better (71). In various cancer types, ICIs-related immunotherapy, particularly PD-1/PD-L1, has shown good therapeutic effectiveness in reversing local immunosuppression in the TME. For tumor patients with significant immune cell infiltration but compromised immunity, such as those in our study’s low-risk score group, PD-1/PD-L1 inhibitors are appropriate. The IMvigor210 dataset was utilized as an external anti-PD-L1 cohort for this study to see if our created Tregs-related risk score can predict patient response to anti-PD-L1 medication. When compared to patients in the high-risk scores group, we discovered that patients in the low-risk scores group had substantial clinical and survival advantages. Finally, we evaluated how well Tregs-related risk scores predicted the effectiveness of sorafenib in patients with HCC. Patients with low risk scores performed better with sorafenib, according to our research. Anti-PD1/PD-L1 with sorafenib may be a viable option for HCC patients in the low-risk score category to improve their prognosis.

The efficacy and prognosis of HCC patients receiving immunotherapy are significantly impacted by drug resistance, which is mostly caused by the complexity and diversity of TME components. Promising treatment now involves reducing the number of tumor-associated macrophages (TAMs) by preventing monocyte recruitment to the TME, eliminating invading TAMs, or re-educating TAMs to the more pro-inflammatory M1 subtype (72). By attracting macrophages and Tregs to the TME of HCC, tumor-associated neutrophils (TANs) cause sorafenib resistance in HCC patients, encouraging cancer development and post-treatment recurrence (73). Targeting Tregs may modify TME composition and speed tumor remission, but it may also result in significant systemic autoimmunity and inflammation (74). Immune-checkpoint molecules, such as PD1, are expressed by tumor-infiltrating Tregs at levels that rely on the TME, suggesting that PD1 inhibitors may have an impact on Tregs infiltration (75). It is clear that PD1 signaling lessens the immunosuppressive effect of Tregs since PD1-deficient Tregs or PD1 inhibition had enhanced immunosuppressive activity that was sufficient to reverse the auto-immune phenotype (76). Furthermore, human glioblastoma tissues with high PD1 expression levels in Tregs have a fatigued phenotype, which is linked to diminished immunosuppressive activity (77). When PD1 on Tregs interacts directly with PD-L1 on CD8^+^ T cells, immunosuppressive effects are directly mediated, and PD1 inhibitors can drastically reduce these effects (78). Monitoring tumor-infiltrating Tregs alterations in patients taking PD1 inhibitors may be important since PD1 appears to have a detrimental influence on Tregs-mediated immunosuppression in tumors and anti-PD1 medication appears to increase Tregs activity (79). For HCC patients, comprehensive immunotherapy targeting both tumor cells and immunosuppressive cells in the TME may become the treatment of choice, and it will likely play a significant guiding role in the choice of patient-specific immunotherapy regimens in the future.

Undoubtedly, our study has certain flaws. First off, it was hard to fully investigate the effect of our Tregs-related signature on the prognosis of HCC patients due to the small number of HCC tissues we gathered and the paucity of survival data. To verify the precision of our prognostic model, we require a prospective multicenter investigation with a bigger sample size. Additionally, the outcomes of single-cell sequencing can aid in our understanding of how the Tregs-related genes have changed in the TME. Finally, to further understand the molecular processes by which Tregs-related genes influence HCC development, functional tests (*in vitro* and *in vivo*) should be carried out in the future.

## Conclusions

In summary, our study uncovered and validated a Tregs-related prognostic model that could identify TME- exhausted subpopulations and revealed that PD1/PD-L1 inhibitors could alter the expression levels of HMOX1, MMP9, and TNFRSF11B in Hep3B and Huh7 cells, which might help us better understand Tregs infiltration and develop personalized immunotherapy treatments for HCC patients.

## Data availability statement

We can find the datasets analyzed in this study at the https://xena.ucsc.edu, https://dcc.icgc.org/projects/LIRI-JP, and https://www.ncbi.nlm.nih.gov/geo. The accession number(s) can be found in the article/**Supplementary Material**.

## Ethics statement

The studies involving human participants were reviewed and approved by the ethics committees of Zhengzhou University. The patients/participants provided their written informed consent to participate in this study. Written informed consent was obtained from the individual(s) for the publication of any potentially identifiable images or data included in this article.

## Author contributions

GZ designed and experimentally validated this study.

## Conflict of interest

The author declares that the research was conducted in the absence of any commercial or financial relationships that could be construed as a potential conflict of interest.

## Publisher’s note

All claims expressed in this article are solely those of the authors and do not necessarily represent those of their affiliated organizations, or those of the publisher, the editors and the reviewers. Any product that may be evaluated in this article, or claim that may be made by its manufacturer, is not guaranteed or endorsed by the publisher.
