# Supplementary material for: Regulatory T-cells-related signature for identifying a prognostic subtype of hepatocellular carcinoma with an exhausted tumor microenvironment
Source: Front Immunol. 2022 Sep 15;13:975762. doi: 10.3389/fimmu.2022.975762 (PMC9521506; doi:10.3389/fimmu.2022.975762)
Supplement: Supplementary file 1 [file DataSheet_1.pdf]

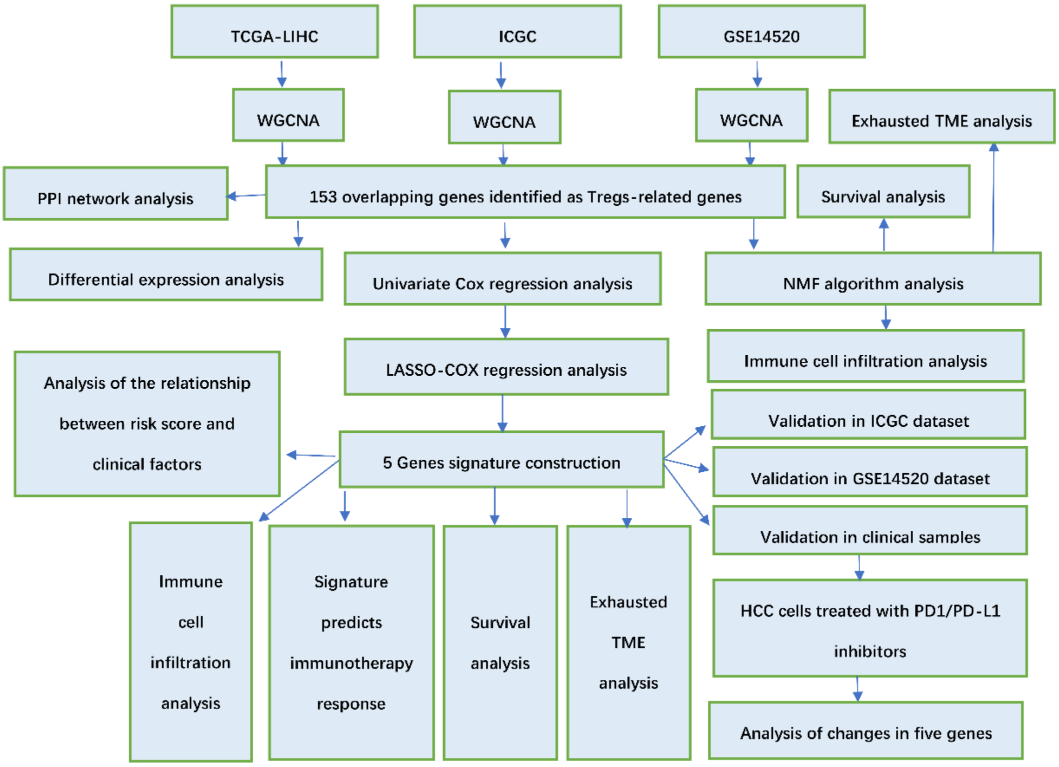

Figure S1 The flowchart.

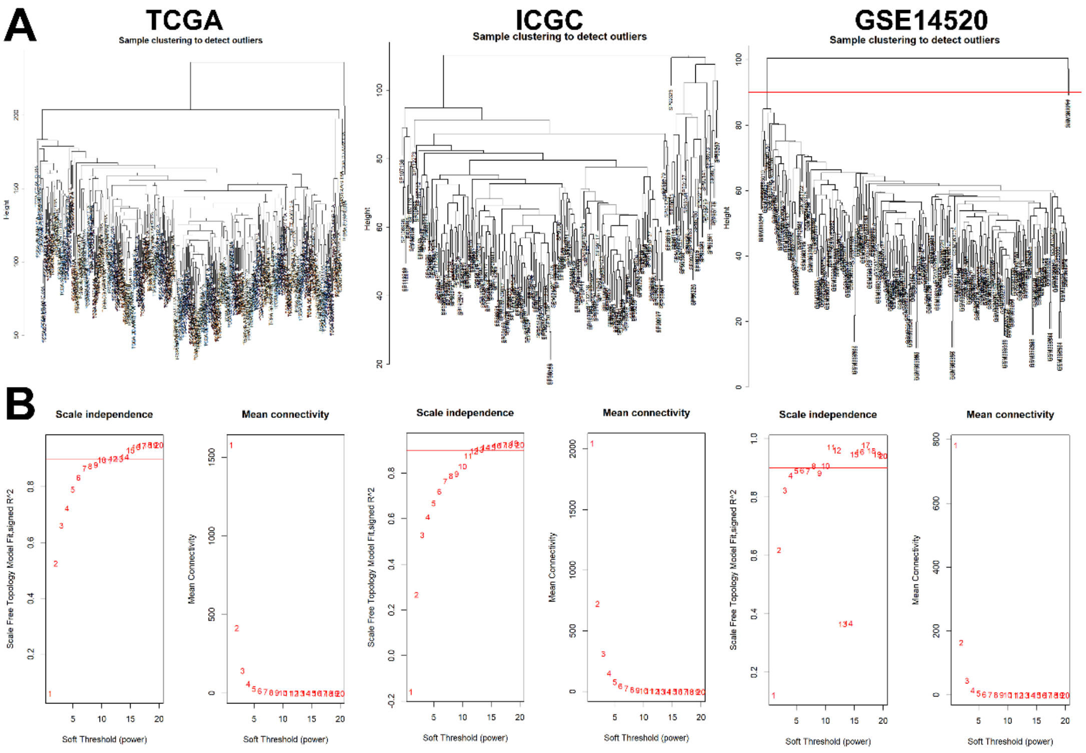

Figure S2 WGCNA analysis in the TCGA, the GSE14520, and the ICGC cohorts. (A) Screening of samples for WGCNA analysis. (B) Identify the

weighted value β that meets the law of scale-free networks.

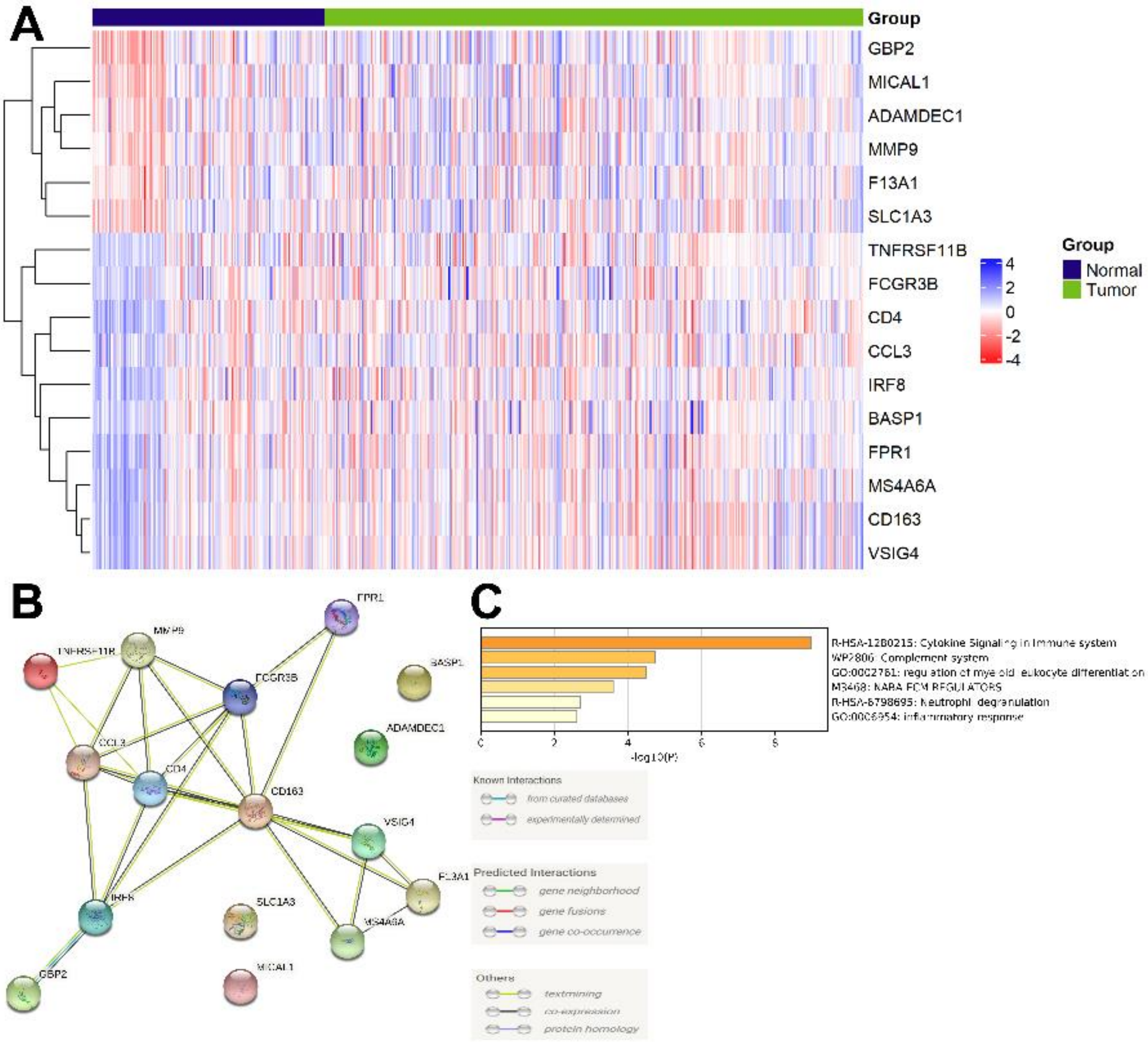

Figure S3 (A) Expression of 16 Tregs-related DEGs in normal and HCC tissues. (B) PPI network was performed to explore the potential interactions between these DEGs. (C) The biological importance of these Tregs-related DEGs were mainly enriched in Cytokine signaling and the regulation of leukocytes.

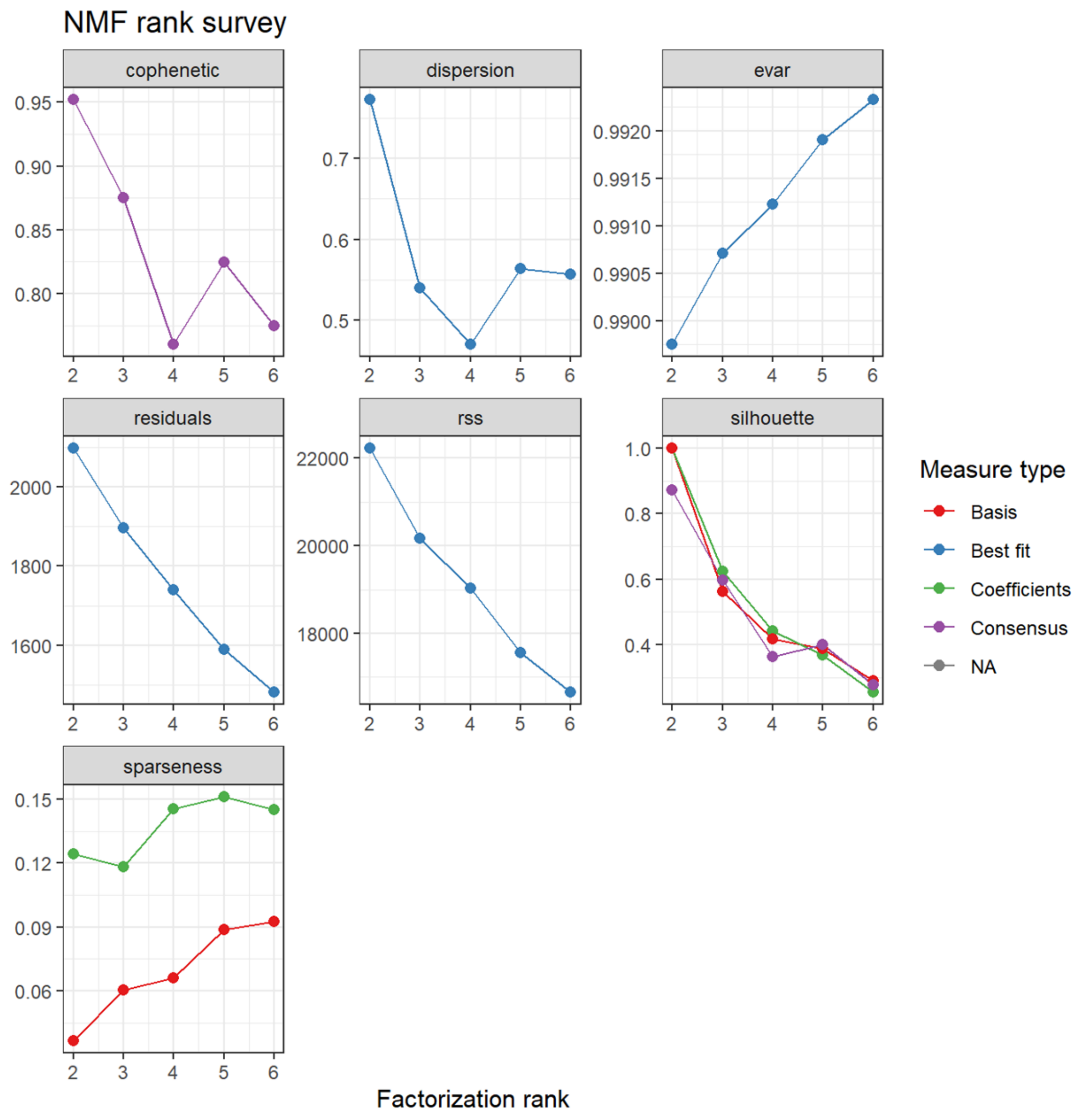

Figure S4 The cophenetic, dispersion, and profile of the NMF algorithm.

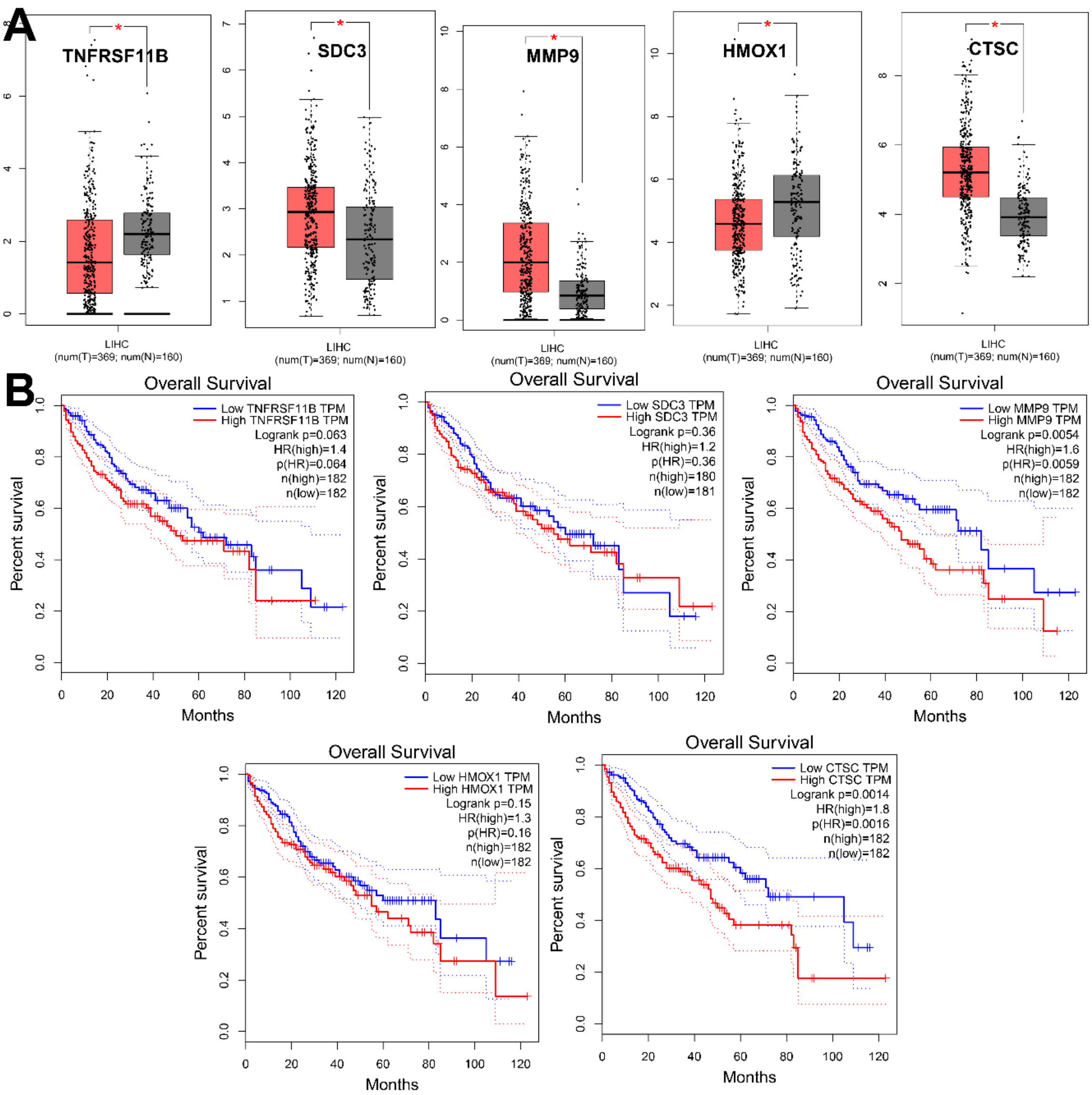

Figure S5 Expression levels (A) and prognostic significance (B) of the five genes in normal and HCC tissues explored in GEPIA. *p < 0.05.

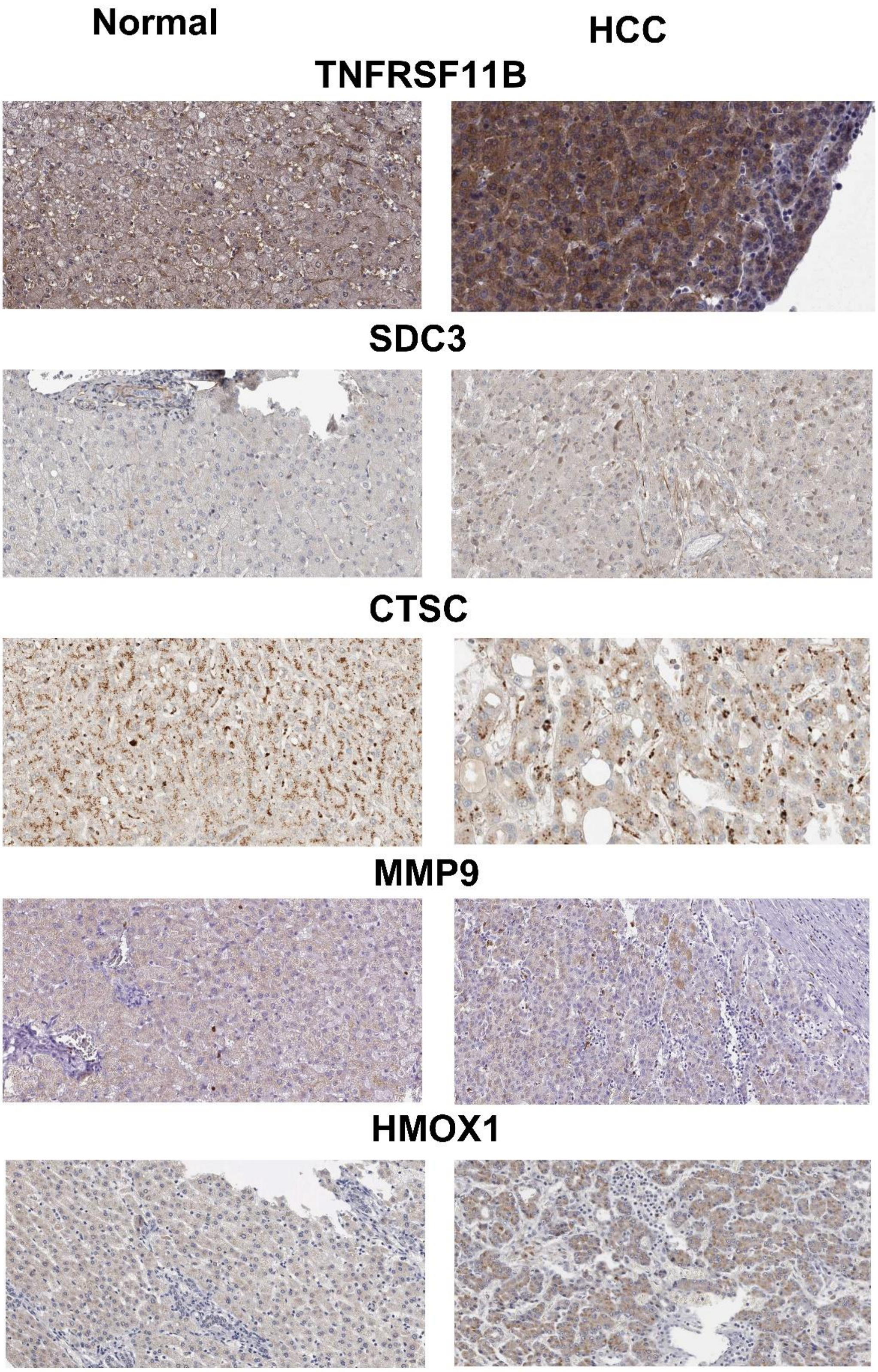

Figure S6 Expression levels of the five genes in normal and HCC tissues explored in HPA.

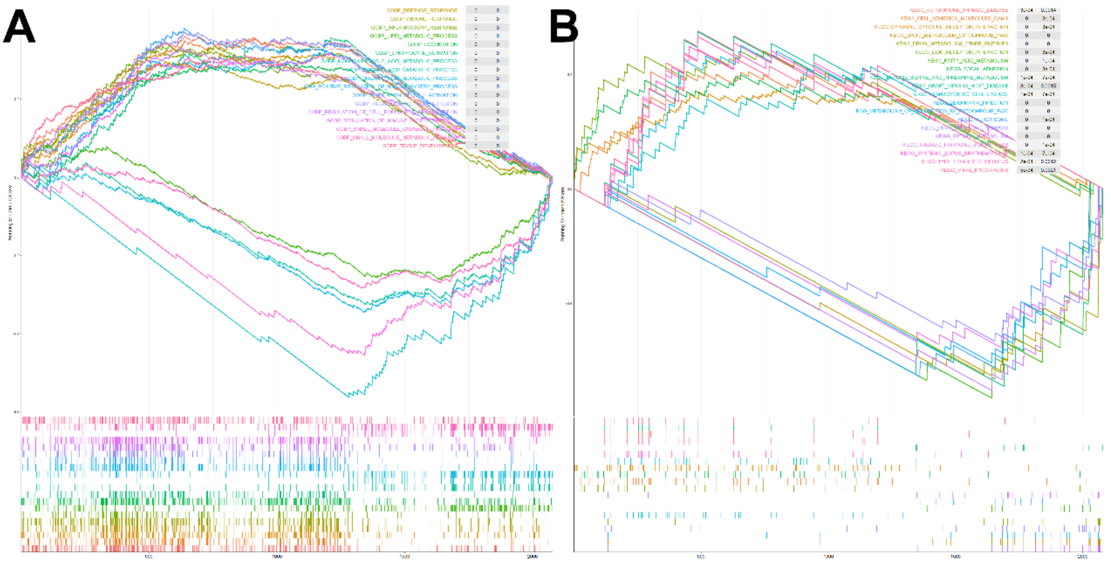

Figure S7 Identification of altered GO and KEGG items between patients in high- and low-risk scores groups.

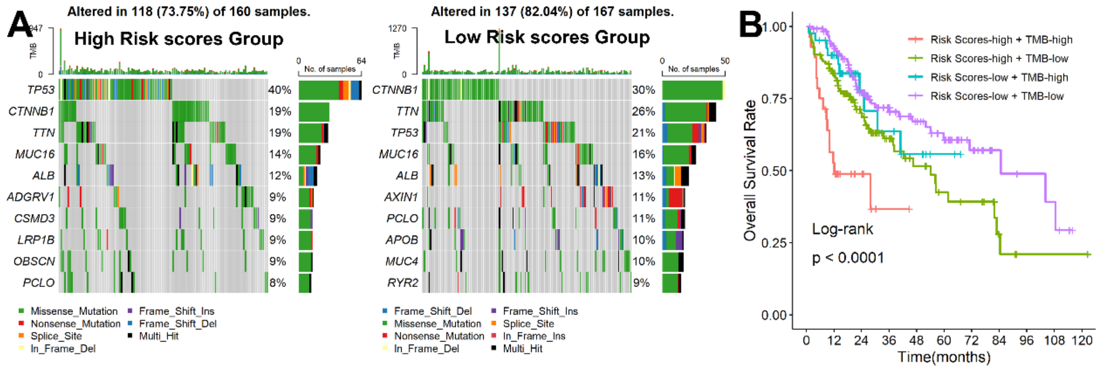

Figure S8 Genetic alterations analysis. (A) Tumor maps of top ten mutated genes between patients in high- and low-risk scores groups. (B) Patients with high TMB levels who had high risk scores had the lowest survival rates.

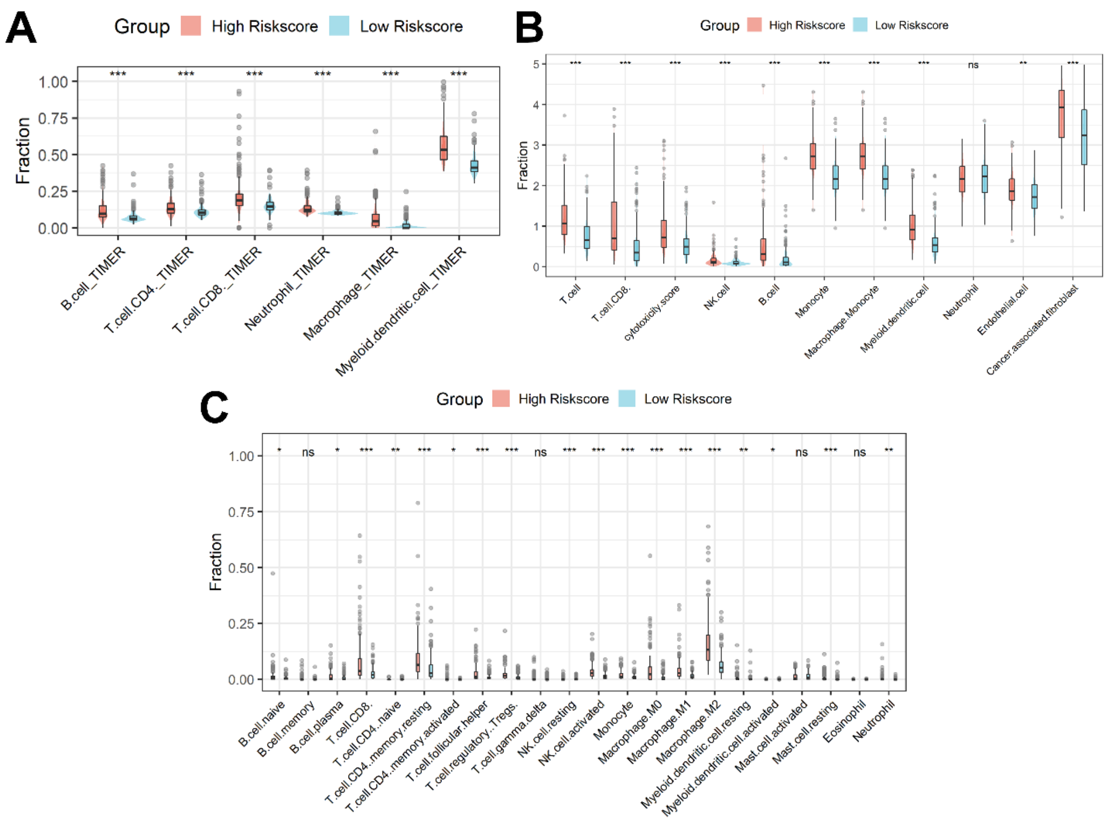

Figure S9 The abundance of immune cell infiltration in HCC patients was assessed using various methods including TIMER (A), MCP-counter (B), and CIBERSORT (C) algorithms. *p < 0.05; **p <0.01; ***p < 0.001

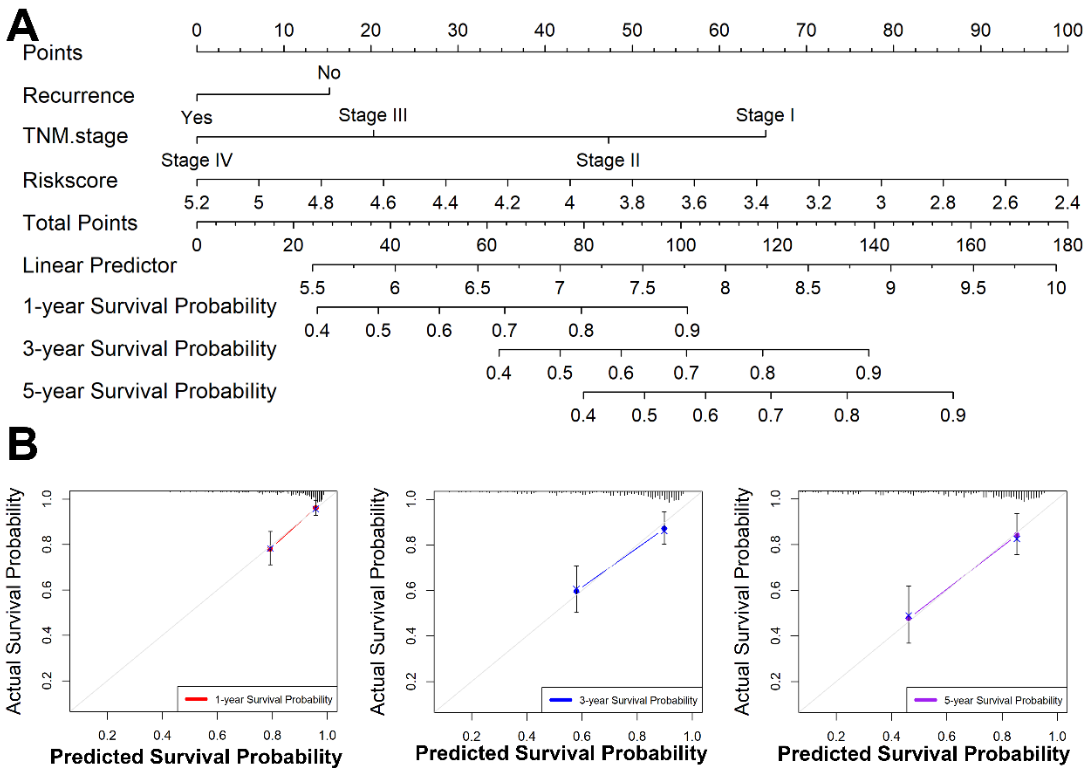

Figure S10 (A) A nomogram model was built in the TCGA dataset to investigate the coefficient prediction efficiency of this Tregs-related signature. (B) The calibration curves showed good agreement between the anticipated and actual probability of 1-, 2-, 3-, and 5-year survival rates.

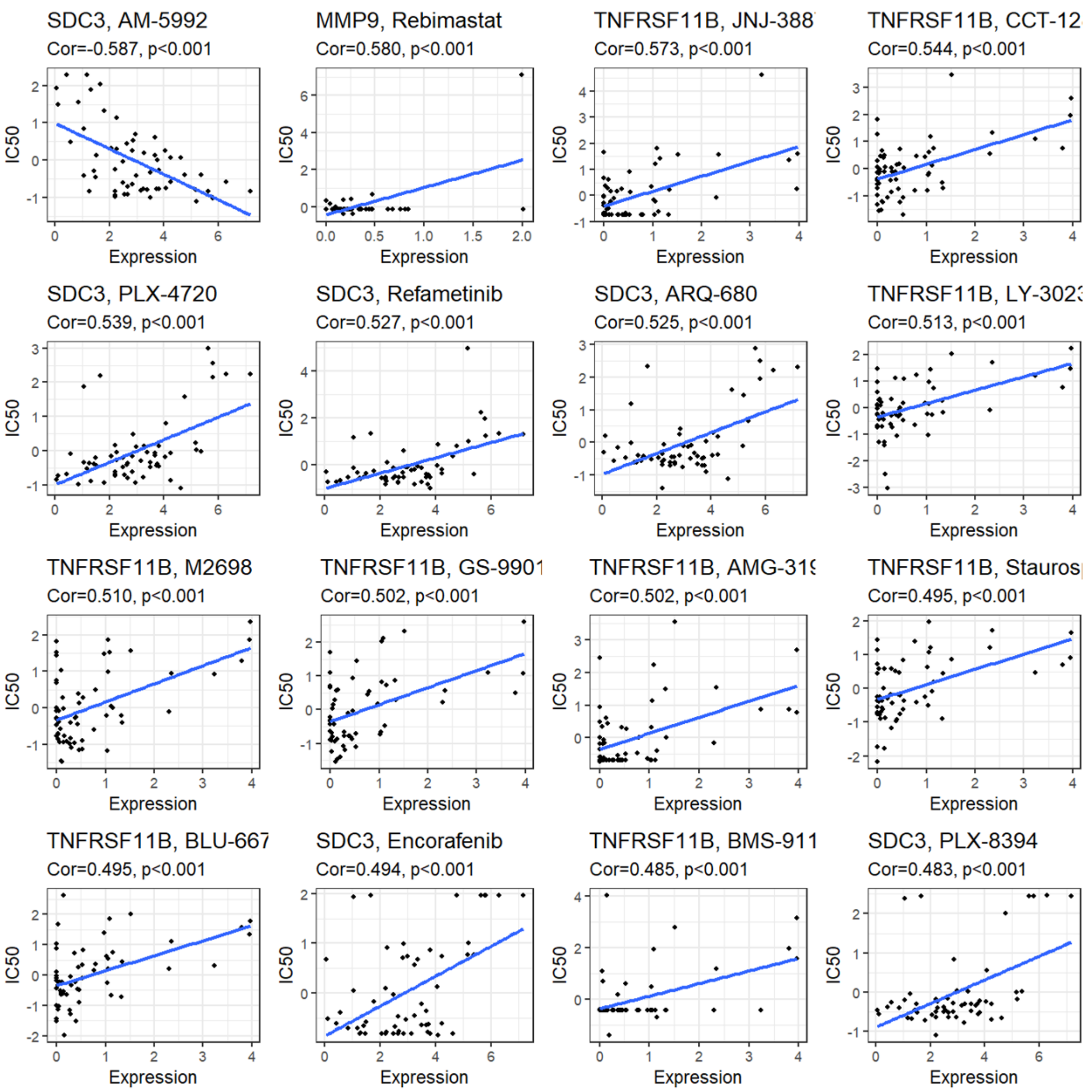

Figure S11 Top 16 most important tumor-sensitive drugs.

Table S1 Clinical characteristics of HCC patients involved in the study

		TCGA cohort (N=342)	ICGC cohort (N=227)	GSE14520 cohort (N=214)
**Gender**	**Male**	233	61	185
	**Female**	109	166	29
**Age**	**≤60 years**	165	49	176
	**>60 years**	177	178	38
**Grade**	**G1/2**	214		
	**G3/4**	123		
	**unknown**	5		
**TNM Stage**	**I/II**	238	140	165
	**III/IV**	83	87	49
	**unknown**	21	0	0
**Vascular Invasion**	**Yes**	101		
	**No**	187		
	**unknown**	54		
**Recurrence**	**With tumor**	122		119
	**Tumor free**	153		95
	**unknown**	67		
**Cirrhosis**	**With**	65		196
	**Without**	134		18
	**unknown**	143		0
**HBV or HCV Infection**				
	**Yes**	141		208
	**No**	161		6
	**unknown**	40		0

Table. S2 The sequences of the qRT-PCR primers used in this study

Gene	Forward primer	Reverse primer
**HMOX1**	AAGACTGCGTTCCTGCTCAAC	AAAGCCCTACAGCAACTGTCG
**MMP9**	GGGACGCAGACATCGTCATC	TCGTCATCGTCGAAATGGGC
**CTSC**	GTTCCCGAAGCGACATTAACT	TCGTAGGCAGTATCCAACTTCTT
**SDC3**	TGGCGCAGTGAGAACTTCG	CCCCGAGTAGAGGTCATCCAG
**TNFRSF11B**	GTGTGCGAATGCAAGGAAGG	CCACTCCAAATCCAGGAGGG
**CD8A**	ATGGCCTTACCAGTGACCG	AGGTTCCAGGTCCGATCCAG
**GZMB**	CCCTGGGAAAACACTCACACA	GCACAACTCAATGGTACTGTCG
**IFNG**	TCGGTAACTGACTTGAATGTCCA	TCGCTTCCCTGTTTTAGCTGC
**TBX2**	CCCCTTCAAGGTGCGAGTC	TCAGCGGCTACAATGTCCATC
**TNF**	CCTCTCTCTAATCAGCCCTCTG	GAGGACCTGGGAGTAGATGAG
**PD1**	CCAGGATGGTTCTTAGACTCCC	TTTAGCACGAAGCTCTCCGAT
**PD-L1**	TGGCATTTGCTGAACGCATTT	TGCAGCCAGGTCTAATTGTTTT
**β-ACTIN**	CGTGGGCCGCCCTAGGCACCA	TTGGCTTAGGGTTCAGGGGGG

Table S3 Tumor-sensitive drugs targeting Tregs-related prognostic model genes.

Gene	Drug	Cor	pvalue	Gene	Drug	Cor	pvalue
SDC3	AM-5992	-0.58713317	1.02E-06	TNFRSF11B	AZD-5363	0.456932	0.000275
MMP9	Rebimastat	0.579939783	1.48E-06	SDC3	CC-90003	0.452169	0.000324
TNFRSF11B	JNJ-3887618	0.572773436	2.13E-06	SDC3	LY-3009120	0.450501	0.000343
TNFRSF11B	CCT-128930	0.543677405	8.59E-06	SDC3	umbralisib	-0.4485	0.000367
SDC3	PLX-4720	0.538748299	1.07E-05	TNFRSF11B	JNJ-38877605	0.446972	0.000387
SDC3	Refametinib	0.527186648	1.79E-05	SDC3	Dexrazoxane	-0.44359	0.000433
SDC3	ARQ-680	0.525295566	1.94E-05	TNFRSF11B	TAS-115	0.438984	0.000505
TNFRSF11B	LY-3023414	0.513288463	3.22E-05	SDC3	MLN-2480	0.437319	0.000533
TNFRSF11B	M2698	0.510136669	3.67E-05	TNFRSF11B	IDH-C227	0.436097	0.000555
TNFRSF11B	GS-9901	0.502449554	5.01E-05	TNFRSF11B	SAR-260301 (enantiomer 1)	0.43567	0.000563
TNFRSF11B	AMG-319	0.501585602	5.19E-05	SDC3	Hydroxyurea	-0.43369	0.0006
TNFRSF11B	Staurosporine	0.495177054	6.69E-05	SDC3	ON-123300	-0.431	0.000655
TNFRSF11B	BLU-667	0.494870015	6.77E-05	TNFRSF11B	VS-5584	0.429395	0.00069
SDC3	Encorafenib	0.493786193	7.06E-05	SDC3	CEP-32496	0.426235	0.000763
TNFRSF11B	BMS-911543	0.484818467	9.97E-05	SDC3	CCT-251545	-0.42327	0.000837
SDC3	PLX-8394	0.482801074	0.000108	SDC3	BOS-172722	-0.42231	0.000863
SDC3	Vemurafenib	0.476244997	0.000137	TNFRSF11B	AT-13148	0.418037	0.000986
TNFRSF11B	SAR-245409	0.476172643	0.000138	TNFRSF11B	dimethylfasudil	0.418012	0.000986
SDC3	Palbociclib	-0.47502157	0.000144	SDC3	CX-5461	-0.4172	0.001011
TNFRSF11B	SGX-523	0.473701667	0.000151	HMOX1	Telatinib	0.416039	0.001048
SDC3	P-529	0.469998732	0.000173	TNFRSF11B	RG-7440	0.414907	0.001085
SDC3	TAK-632	0.464077455	0.000214	SDC3	JNJ-54302833	-0.41434	0.001104
TNFRSF11B	IPI-145	0.46398267	0.000214	TNFRSF11B	enantiomer of PF-4176340	0.412042	0.001184
TNFRSF11B	Idelalisib	0.460868671	0.000239	TNFRSF11B	Afuresertib	0.412032	0.001185
SDC3	Dabrafenib	0.458696146	0.000258	MMP9	Triapine	0.410839	0.001228
SDC3	SB-590885	0.457411791	0.00027	TNFRSF11B	AZD-8186	0.408649	0.001312
SDC3	KPT-8602	-0.40002857	0.001694	SDC3	PD 184352	0.405244	0.001453
